# Genetically engineered probiotic for the treatment of phenylketonuria (PKU); assessment of a novel treatment *in vitro* and in the *PAH^enu2^* mouse model of PKU

**DOI:** 10.1371/journal.pone.0176286

**Published:** 2017-05-17

**Authors:** Katherine E. Durrer, Michael S. Allen, Ione Hunt von Herbing

**Affiliations:** 1 Department of Biological Sciences, University of North Texas, Denton, Texas, United States of America; 2 Institute of Molecular Medicine, Center for Medical Genetics, University of North Texas Health Science Center, Fort Worth, Texas, United States of America; University of Illinois at Urbana-Champaign, UNITED STATES

## Abstract

Phenylketonuria (PKU) is a genetic disease characterized by the inability to convert dietary phenylalanine to tyrosine by phenylalanine hydroxylase. Given the importance of gut microbes in digestion, a genetically engineered microbe could potentially degrade some ingested phenylalanine from the diet prior to absorption. To test this, a phenylalanine lyase gene from *Anabaena variabilis* (AvPAL) was codon-optimized and cloned into a shuttle vector for expression in *Lactobacillus reuteri* 100-23C (pHENOMMenal). Functional expression of AvPAL was determined *in vitro*, and subsequently tested *in vivo* in homozygous *PAH*^*enu2*^ (PKU model) mice. Initial trials of two *PAH*^*enu2*^ homozygous (PKU) mice defined conditions for freeze-drying and delivery of bacteria. Animals showed reduced blood phe within three to four days of treatment with pHENOMMenal probiotic, and blood phe concentrations remained significantly reduced (P < 0.0005) compared to untreated controls during the course of experiments. Although pHENOMMenal probiotic could be cultured from fecal samples at four months post treatment, it could no longer be cultivated from feces at eight months post treatment, indicating eventual loss of the microbe from the gut. Preliminary screens during experimentation found no immune response to AvPAL. Collectively these studies provide data for the use of a genetically engineered probiotic as a potential treatment for PKU.

## Introduction

Affecting 1 in 15,000 people worldwide, phenylketonuria (PKU) most commonly occurs when any two of the known 700+ loss-of-function mutations in the gene for the hepatic enzyme PAH (phenylalanine hydroxylase) are inherited by a patient. Without PAH to convert phenylalanine (phe) into tyrosine, phe and its alternative metabolites accumulate to neurotoxic levels in the blood. One of these metabolites, phenylketone, is excreted in the urine giving the condition its name. Untreated PKU results in severe neurological damage including tremors, seizures, and mental retardation. Treated patients may have minor secondary conditions accompanying PKU including mood disorders [1], minor cognitive defects [2], early onset osteopenia [3, 4], and markers of systemic inflammation [3]. Additionally, pregnant women with PKU must maintain very low blood phe levels (120–360μM) to prevent cardiac and neurological birth defects in their infants (maternal PKU) [5].

The only effective treatment for all PKU patients is restriction of dietary phe. As an essential amino acid, phe cannot be removed from the diet and no natural proteins are phe free. Synthetic medical foods and supplements for the low phe diet are available but can be expensive and are not universally covered by patient insurance. Medical foods and supplements also require more time to prepare compared to ordinary foods, and are reported as having unfavorable flavor and odor [6]. As a result, many PKU patients relax or abandon the restrictive diet upon leaving their parents (pers. com. NSPKU and NPKUA 2012–2014). Development of new technology is therefore desired to provide less expensive and more patient friendly treatments.

Past oral phenylalanine ammonia lysase (PAL) studies required the protection of PAL (naked enzyme or enzyme within cells) from stomach acid in the form of antacid buffers, but indicated enzymatic digestion of phe in the intestine could lower blood phe levels in the PKU mouse [7, 8]. Additional studies indicated rapid cleavage of PAL enzymes by intestinal proteases should an enteric delivery of naked PAL be attempted [9]. Mimicking the known ability of gut bacteria to assist in host digestion [10, 11], a PAL gene expressed within an orally administered probiotic would be able to metabolize some of the phe ingested by the host.

*Lactobacillus reuteri* is a well studied probiotic bacteria with strains deemed safe for human [12, 13] and mouse [14–17] use. Orally administered lactobacilli survive the stomach and proliferate in the intestine where they are metabolically active. Moreover the systemic immune response observed in PEG-PAL [9, 18, 19] will not be observed in a treatment of PAL expressed within lactobacilli, as gut microbes and their proteins/enzymes participate in a phenomenon known as immunological ignorance [20]. Using *L*. *reuteri* as a delivery system, PAL will reach the intestine intact to catabolize phe present in the intestinal lumen reducing phe entering the blood stream from the diet.

For this study rodent specific *Lactobacillus reuteri* 100-23C was genetically engineered to express PAL enzyme consistent with that of *Anabaena variabilis* ATCC 29413 (AvPAL). AvPAL was selected for insertion into the *L*. *reuteri* 100-23C based on its high processivity, low degradation rate in foreign bacterial hosts, small size, substrate specificity, and ability to fold without chaperone proteins [21]. The resulting product has been named "pHENOMMenal". This study had two objectives: 1) to engineer a probiotic with *in vitro* phe catabolism, and 2) to verify the ability of the probiotic to function as an enzyme replacement therapy *in vivo* using *PAH*^*enu2*^ mouse model of PKU. The results support a potential therapeutic option for engineered probiotics to treat metabolic diseases such as PKU.

## Materials and methods

### Growth and transformation of Top 10 *E*. *coli*

Top 10 Chemically Competent *E*. *coli*, F- *mcrA* Δ (*mrr-hsdRMS-mcrBC*) Φ*80lac*ZΔ*M15* Δ *lacX74 recA1 araD139* Δ (*araleu)7697 galU galK rpsL (StrR) endA1 nupG*, were used for plasmid propagation (Life Technologies, USA). Top 10 *E*. *coli* cells were grown in Luria Broth (LB) (MoBio, USA) and Terrific Broth (TB) (MoBio, USA) or on Luria agar plates as required by the protocol. Plates were incubated at 37°C in a standard aerobic incubator, liquid cultures in a 37°C aerobic shaker. Antibiotic was added as appropriate for the selected plasmid (pSLER1, pSLERGT) at concentrations of 50μg/mL ampicillin or 300μg/mL erythromycin. Transformation of Top 10 chemically competent cells performed according to strain manual.

### Rosetta *E*. *coli* transformation, growth and protein purification

Rosetta 2 DE3 chemically competent *E*. *coli*, F- *ompT hsdS*_*B*_*(r*_*B*_^*-*^*m*_*B*_^*-*^*) gal dcm (DE3) pRARE2(CAM*^*R*^*)*, (Novagen Millipore, USA) were transformed with p-HIS8-AvPAL plasmid (a gift from Dr. Bradley Moore, UC San Diego) as directed by the cell strain manual. A successful transformant growing on an LB agar plate with 20μg/mL kanamycin and 35μg/mL Chloramphenicol was cultured in TB with 20μg/mL kanamycin and 35μg/mL Chloramphenicol at 37°C in an aerobic shaker to produce a final OD_600_ of 0.600 for use in creating culture stock. Stock vials were made using 50μl 10% glycerol in 1xPBS, and 50μl of the above liquid culture for storage at -80°C. 10μl of frozen Rosetta *E*. *coli* (Rosetta cells hereafter) stock carrying the p-HIS8-AvPAL plasmid were added to a flask with 50ml of TB with 20μg/mL kanamycin and 35μg/mL Chloramphenicol to shake at 225rpm aerobically 37°C for 18 hours. An aliquot of these cultured Rosetta cells were transferred into 250ml fresh TB with above antibiotic concentrations for a new OD_600_ = 0.100 and grown for 3–4 hours at 225rpm and 37°C to reach an OD_600_ = 0.600. Once at this OD_600_, isopropyl β-D-1-thiogalactopyranoside (IPTG) was added to induce protein expression at a final concentration of 100mM IPTG then shaken at 225rpm 23°C for 18 hours.

Protein extraction from the IPTG induced Rosetta cells was performed by affinity purification using B-PER 6xHis Fusion Protein Purification kit (Pierce Protein Products, USA) or the reagents of this kit with Amicon Pro Purification Columns (Millipore, USA). When necessary, extracted protein was concentrated with an Amicon Ultra Concentration Column (Millipore, USA) per manufacturer's directions. Purified AvPAL protein was run through High-Capacity Endotoxin Removal Resin spin columns (Pierce Protein Products, USA) per manufacturer’s directions in 4–5 serial removal protocols/columns to reduce endotoxin contamination of the sample to acceptable levels as determined by Limulus Amebocyte Lysate (LAL) assay Chromogenic Endotoxin Quantitation Kit (Pierce Protein Products, USA). Final protein concentration was determined by Bradford assay, using BSA pre-mixed standards (Pierce Protein Products, USA), and Coomassie blue stain in 96well UV transparent plates in the Synergy 2 plate reader detecting absorbance at 595nm.

### Growth and transformation of *Lactobacillus reuteri* 100–23 strains

*Lactobacillus reuteri* 100–23 strains were grown using deMan, Rogosa and Sharpe (MRS) broth (Fisher Scientific, BD Difco, USA) and MRS agar (Fisher Scientific, BD Diffco, USA). *L*. *reuteri* 100-23C [22] (a gift from Dr. Gerald Tannock, University of Otago, New Zealand) contains no plasmids, and was grown in plain MRS. *L*. *reuteri* 100–23 strains with erythromycin resistance were grown in with 5μg/ml of erythromycin in MRS. Agar plated and liquid cultures were incubated at 37°C in anaerobic jars using gas packs (Thermo Scientific Oxoid, USA) to generate anaerobic conditions. Anaerobic indicator slips (Thermo Scientific Oxoid, USA) verified O_2_ absence. Liquid cultures were grown 12–18 hours, plated cultures 48 h. To produce frozen stock of various *L*. *reuteri* 100–23 strains, liquid cultures were aliquoted as 50μl culture from the 12–18 hour growth with 50μl of sterile 10% skim milk and stored at -20°C or -80°C.

For transformation *L*. *reuteri* 100–23 cells were grown (37°C anaerobic) to an optical density at 600 nm (OD_600_) of 0.8–1.0 and harvested by centrifugation (9,820 xg for 10 minutes at 4°C) and media decanted. Cells were washed twice with ¼ original volume sterile 3.5X SMEB (952 mM sucrose, 3.5 mM MgCl_2_, adjusted to pH 7.2) with repeats of the centrifugation and liquid removal after each wash. The cell pellet was re-suspended in 1/20^th^ original volume chilled sterile 3.5X SMEB. 400 μl of cell suspension and 1.0 μg plasmid DNA were added to a 0.2 cm electroporation cuvette on ice. GenePulser (BioRad, USA) subjected cells to a pulse of 12.5 kV/cm, 200 ohm parallel resistance, 25 μFD capacitance. Cells were immediately transferred to 10ml pre-warmed sterile MRS and incubated 3 h at 37°C, aerobic. Cells were centrifuged (7,740 xg, 5 minutes, 4°C) and re-suspended in 500 μl of MRS, diluted and plated for colony positive clone isolation.

### Growth to harvest *L*. *reuteri* 100–23 cells for freeze drying

10μl of the desired frozen *L*. *reuteri* 100–23 stock was added to a 50ml conical tube with 40mL MRS with 5μg/ml ery and allowed to grow for 18 hours. OD_600_ was assessed after growth and cells were diluted into multiple flasks of 600ml fresh MRS (5μg/ml ery) to an OD_600_ of 0.05–0.150 for the second growth phase at 37°C in anaerobic jars and gas packs (Oxoid, USA). Once the second growth reached an OD_600_ between 0.575 and 0.800, cells were placed on ice for 10 min to stop growth.

### CFU quantitation of freeze dried *L*. *reuteri* 100–23 cells

A small aliquot of freeze dried powder (FDP) *L*. *reuteri* 100–23 cells was suspended in sterile chilled PBS at a rate of 0.001g FDP/ml PBS to make the starting suspension. This suspension was serially diluted (10x) in MRS with 5μg/ml ery. For agar plate counting; dilutions of 10^−3^ through 10^−6^ were used when triplicate plating 100μl of dilution on MRS agar plates with 5μg/ml ery and incubated at 37°C anaerobically (see above) for 48h. For liquid MRS in 96 well plate counting; dilutions of 10^−3^ through 10^−8^ were plated with ten replicates per dilution when using 70μl of each dilution with 180μl MRS with 5μg/ml ery per well and grown at 37°C anaerobically (see above) for 24h.

### Creation of pSLERGT and insertion of synthetic AvPAL gene

pNCKH103 plasmid [17] (a gift from Drs. Gerald Tannock and Nicholas Heng, University of Otago, New Zealand) harvested from transformed *L*. *reuteri* 100–23 cells served as template for extension PCR of the pGT232 fragment using 6μl 10x Q5 polymerase reaction Buffer, 1.5μl of 15ng/μl total DNA including pNCKH103 DNA from *L*. *reuteri* 100–23, 1.5μl of 10μM Forward Primer 5’tagctgagtcgacaacagttgttaa 3’, 1.5μl of 10μM Reverse Primer 5’gagagaataaatcctccatggtttcttaga 3’, 2.4μl 10mM dNTP, 18.6μl Molecular water, 0.25μl Q5 DNA polymerase (New England Biolabs, USA). Thermocycler conditions: initial denaturation 98.0°C 30 s; 4 cycles of 98°C 10 s, 57°C 12 s, 72°C 90 s; 30 cycles of 98°C 10 s, 65°C 12 s, 72°C 90 s; final extension at 72°C for 120 s then hold at 4°C. PCR products were run as a mirror gel and gel purified from the portion without ethidium bromide using the UltraClean^®^ GelSpin^®^ DNA Extraction Kit (MoBio, USA Laboratories, Inc., USA).

This pGT232 fragment was cut using NcoI FastDigest (ThermoScientific, USA) for one sticky and one blunt end. pSLER1 vector [23] (a gift from Dr. Nicholas Heng, University of Otago, New Zealand) was cut using FastDigest enzymes NcoI and SmaI (ThermoScientific, USA) resulting in a sticky/blunt vector. Both digests were run utilizing recommended concentrations from the manufacturer and 90 min reaction time at 37°C. Reactions were each cleaned using the solutions protocol of the UltraClean^®^ GelSpin^®^ DNA Extraction Kit (MoBio, USA Laboratories, Inc.) and eluted into molecular water. 2μl 10x T4 DNA ligase buffer (ThermoScientific), 2μl 50% PEG 4000 (ThermoScientific), 1μl digested pSLER1 at 21ng/μl, 5.5μl digested pGT232 fragment at 6ng/μl, and 8.5μl molecular water were combined for ligation at room temperature for 60 min. 5μl of the ligation reaction was added to 50μl of chemically competent Top10 *E*. *coli* from Life Technologies, USA for transformation (see above).

Colonies with ligated plasmid were selected on LB agar containing 50μg/mL of ampicillin, and further growth for plasmid extraction (UltraClean 6 minute mini prep, MoBio, USA) was performed in TB dry broth containing 300μg/ml of erythromycin. Desired ligation of the plasmid in each colony was determined from extracted plasmid (Ultraclean 6 Minute Mini Plasmid Prep Kit, MoBio, USA) by restriction digest with FastDigest enzymes NcoI and SalI (Thermo Scientific, USA) and visualization on ethidium bromide gel. The clone with successful insertion, pSLERGT, was sequence verified by BioBasic Inc. (Canada).

In order to produce functional enzyme within a Lactobacillus host, the original DNA sequence for PAL from *Anabaena variabilis* ATCC 29413 (AvPAL) was altered in several ways. The indigenous AvPAL promoter was replaced with a Lactobacillus high production constitutive promoter and ribosome binding sequence from the erythromycin resistance B gene (*ermB*)[24]. Codons in AvPAL were optimized (altered) to match Lactobacilli codon usage to increase efficiency of expression in *L*. *reuteri* 100–23. To reduce potential complications caused by the insertion of an AvPAL gene into the system, the transcriptional terminator from the *ermGT* resistance gene (*ermGT*) was added after the AvPAL stop codon [25]. The full construct, *FuzErmAvPAL*, was synthesized, inserted into pSLERGT, and sequence verified by BioBasic Inc (Canada).

### AvPAL function *in vitro*

Trans-cinnamate produced by AvPAL cleavage of phe is detectable by the increased absorbance of light at a wavelength of 280nm [21]. Trans-cinnamate standard solutions were produced by dissolving trans-cinnamate in PBS to the desired concentration. Absorbance measurements for known concentrations of 0 μM, 6.25 μM, 12.5 μM, 25 μM, 50 μM, 100 μM, 125 μM, 250 μM, and 400μM trans-cinnamate in PBS served as a standard curve. Unknown samples were quantified using this curve. A control of plain PBS for each cell line/condition was run to account for other cell processes causing an increased absorbance at 280nm. This plain PBS control was subtracted from the PBS+phe for each sample to subtract non-trans-cinnamate metabolites that could increase the absorbance reading at 280nm. Control and experimental *L*. *reuteri* 100–23 cells were grown separately to the same OD_600_ in 25mL of MRS media at 37°C (OD_600_ 0.600–0.750). Cells were chilled on ice 5 min then centrifuged (9,000xg for 7 min, 4°C). Cells were washed in 5mL sterile chilled PBS and re-pelleted (9,000xg for 7 minutes at 4°C). Supernatant was removed completely, and cells were re-suspended in 500μl sterile chilled PBS for sonication on ice (four cycles, 40V, pulse 5 s on, 10 s off). Post sonication solutions were centrifuged (20,000xg for 15 minutes at 4°C) to separate lysate and solids. 30μl of the appropriate lysate was added to 150μl PBS or 150μl PBS with 12mM phe (final concentrations of 0mM phe and 10mM phe respectively) for a total volume of 180μl/well. Each condition was run in triplicate in a BioTek Synergy 2 plate reader at 37°C using a 96 well UV transparent plate, an absorbance wavelength of 280nm, and absorbance was measured at 5 minute intervals from t = 0 to t = 30. This assay was performed three separate times, each replicate utilizing a fresh growth of bacteria.

### Animal care and usage

*PAH*^*enu2*^ mutant mice on the C57BL6/J background were acquired from Dr. Harding of Oregon Health and Science University. Animals were bred and maintained in the University of North Texas animal facility. Water and standard 5LL2 pelleted mouse chow (LabDiet, USA), 0.79% phe, were available to animals not on experimental diets *ad libitum*. For a short time period the standard chow used was 5P07, 0.67% phe, *ad libitum*. A 12/12 light cycle and temperature of 23°C were maintained at all times. All procedures were approved by the University of North Texas Institutional Animal Care and Use Committee under the protocol number 1202–03. In keeping with the Guide for the Care and Use of Laboratory Animals by the National Institutes of Health, all efforts were made to minimize suffering and distress. Animals with unusable genotypes, that became too old to breed, or reached the end of experimentation were euthanized via CO_2_ asphyxiation followed by cervical dislocation to ensure death, consistent with our approved protocol listed above.

### PCR based *PAH* genotyping of mice

HemoKlen Taq kit (New England Biolabs, USA) was used as needed for genotyping the *PAH* alleles following the manufacturers concentrations and reaction conditions for *PAH* PCR product length and primer sequence. Primer sequences for the amplification of *PAH* were *PAH*^*enu2*^ forward primer of 5’TGCTGCAACCTGGTAATACTGATCC 3’, and *PAH*^*enu2*^ reverse of 5’GAACATTGGAGCTTGATGGAATCC 3’. The product is 616 base pairs, and digestion with restriction enzymes BbsI or BsmAI (Thermo Scientific, USA) used as directed in the appropriate manual. Differential banding patterns are visualized on a gel for genotype determination. If using BbsI, the PKU causative allele of *PAH*^*enu2*^ remains uncut at just over 600 base pairs in length (616) while the wild type *PAH* allele is cut into nearly identical bands at 300 base pairs in length. If using BsmAI, the PKU causative allele of *PAH*^*enu2*^ is cut to produce DNA products at 308, 274, and 34 bases while the wild type *PAH* allele is cut into DNA of 342 and 274 bases.

### Mouse blood collection

Dried heparin tubes were created prior to blood draw by adding 10 IU Heparin (dissolved in water) to each autoclave sterilized microcentrifuge tube. The tubes were then placed into a speed vac rotor to remove all liquid while leaving the heparin behind. Blood was collected by cheek bleed (no more than 125μl) into a dry heparin coated tube and centrifuged at 1,000xg for 10 minutes. Plasma collected from the tube and stored in a clean microcentrifuge tube at -20°C until utilized in the desired assay.

### Plasma phe assay

Quantifying plasma phe for genotyping or experimental data was performed by fluorometric assay [26]. Alterations to total volume from the original protocol were made to accommodate use of a plate reader (Synergy, BioTek2, USA) and 96 well plates (black plastic with black bottom by Corning, USA). Briefly; standards of 0 μM, 180 μM, 300 μM, 600 μM, 1200 μM, and 2400μM were created by spiking 1xPBS + 7.5% Bovine serum albumin with phe. Standards were aliquoted in microcentrifuge tubes and stored at -20°C until needed, and tubes were used a maximum of 3 times before disposal and opening of a new set of standards. Step 1: 10μl of plasma (see above) or phe standard and 10μl of 0.6M Trichloroacetic acid were placed into a microcentrifuge tube to react for 10min, then centrifuged at 13,000xg for 10min. Step 2: 3.3μl of liquid per sample was removed from step 1 tubes and added to a new microcentrifuge tube containing 50μl of solution 2 (50μl Solution 2; 6.25μl 5mM L-leucyl-L-alanine + 12.5μl 30mM Ninhydrin + 31.25μl 600mM Sodium Succinate buffer), one tube per sample. All samples were sealed and placed in a 60°C water bath to react for 2 hours. Samples were briefly cooled on the counter after the reaction period, and were centrifuged to ensure all lid condensate rejoined the total sample volume. Step 3: 40μl of each sample from step 2 were placed into individual wells of the black 96 well plate. Once all samples were loaded, 250μl of solution 3 (250μl Solution 3; 100μl 0.6mM copper sulfate + 150μl 25mM Sodium potassium tartrate) was added to each well with a multi channel pipettor, and a fluorometric read (360/520) was performed by the Synergy2 BioTek plate reader.

### Freeze drying of *Lactobacillus* strains

Up to 3.6 L of the appropriate *L*. *reuteri* 100–23 cell lines grown to an OD_600_ 0.550–0.750 were centrifuged (9,000xg for 15 minutes, 4°C). Supernatant was aspirated, and cells re-suspended in ¼ the original volume of chilled sterile PBS and pelleted again (9,000xg for 10 minutes, 4°C). Final cell pellet was weighed, and 1.5x the pellet weight added of sterile chilled PBS for re-suspension. Cell suspension was spread thin in sterile Petri dishes and placed in a -80°C freezer for one hour. Plates were transferred to freeze drying chamber, temperature of -40°C, and maintained at this temperature for the drying process with a vacuum pressure of 0.080 mBarr for 72 hours. Freeze dried cells were manually crushed into a powder with a glass mortar and pestle (sanitized with 70% ethanol prior to each use), weighed and placed into sterile O-ring gasketed microcentrifuge tubes (Denville Scientific, USA) for storage at -20°C. A small weighed sample (0.10–0.25g) of this was used to determine the number of cfu/gram of freeze-dried powder for each batch (see above).

### Probiotic treatments

To minimize pain and distress, gastric gavage was not used to deliver the probiotic to the animals. Instead, freeze dried probiotic was mixed into powdered mouse chow. Reduction in probiotic cfu count once it was mixed within the chow was minimized by mixing fresh food daily (see above). Initial feeding experiments were conducted for 3–4 days with two animals per experiment. Animals were bled on alternating cheeks at the following time points—prior to treatment, and after 3 or 4 days of treatment (3rd day for females, 4 days for males). Aside from number of animals used and shorter time scale, experimental preparations for preliminary experiments were the same as those listed below for longer experiments.

Groups for the seven and fourteen day experiments began with 3–4 male mice or two female mice between the ages of 12 and 14 weeks old. Some samples were lost due to equipment failure, resulting in only 4 samples with all time points for the fourteen day study. Mice consumed standard chow in powdered form (see above) during experimentation to allow addition of freeze-dried probotic to the chow. Males received 2.0 x10^7^ cfu probiotic/gram chow, while recalculation shows females received 1.0 x 10^7^ cfu probiotic/gram of chow. Powdered chow was administered via mouse feeder shield 148-4-MFS (Unifab Corp, USA) with an inner pedestal (3cm tall x 2 inch diameter autoclavable PVC ring) and shallow food dish (60mm sterile Petri dish) to minimize spillage and soiling. Old chow dishes were removed every 24 hours, and replaced by fresh dishes with fresh chow. Based on consumption studies, 4 grams of chow per mouse were prepared each day with probiotic for all days mice received probiotic treatment. Cages receiving probiotic infused food were maintained with micro-isolator lids to prevent undesirable spread of the microbes. Blood was collected from the female mouse experimental run prior to treatment, after three days of treatment, and after seven days of treatment by rotating which cheek was used for blood collection and by collecting small blood samples; this provided the female samples for the 3–4 day initial assessment as well as the seven day assessment point and this is reflected in their matching numbers in the appropriate graphs of these data. Three runs of the experiment were conducted with males. For male animal seven and fourteen day experiments mice were bled prior to treatment, fed regularly for 3 days, then fed control probiotic for four days and bled again. Males were then switched from control probiotic to the pHENOMMenal treatment probiotic and bled after seven fourteen days of treatment with pHENOMMenal. A final blood sample was collected in males treated for fourteen days at four months post treatment for follow up immunogenicity studies. All but the last blood sample collected was analyzed for phe content as described above. Some sample aliquots for the fourteen day time point were compromised, resulting in a mixture of data from two independent runs pooled for the graph shown. Aliquots for Anti-AvPAL IgG assessment were available from two independent experimental runs; data is only shown on the animals matching the fourteen day graph of phe values for consistency purposes. Assessment of permanent pHENOMMenal colonization was performed by collecting fecal samples from the animals in the third male experimental run at four months and eight months post treatment and allowing growth under conditions that would allow *E*. *coli* (LB + ampicillin, aerobic) or *L*. *reuteri* 100–23 (MRS + erythromycin, anaerobic) to grow if they retained the pHENOMM plasmid.

### Anti-AvPAL antibody detection by quantitative ELISA

AvPAL protein with his-tag was produced in Rosetta *E*. *coli* as described above. Endotoxin levels in purified AvPAL samples were confirmed with LAL Chromogenic Endotoxin Quantitation kit (Pierce Protein Products, USA) as less than 10ng/ml endotoxin for coating 96 well plates for ELISA. AvPAL for injection into positive control animals contained less than 2.5ng of endotoxin per weekly injection. Positive control animals received 30μg or 150μg AvPAL via intraperitoneal (IP) injection once weekly for 3 weeks.

For the ELISA, 96 well transparent plates with 100μl per well of 10μg/ml AvPAL were sealed and incubated at 4°C for 18 hours. 7.5% sodium caseinate was added for 15 min to block any unbound well surface area. Wells were washed with wash solution (1xPBS X% Tween-20) 3x and a rinse (1xPBS). 100μl/well of antibody standard (6x-His Epitome Tag Antibody an anti-mouse IgG2b monoclonal antibody, Pierce Protein Products USA) or diluted mouse plasma were added for incubation at room temp, 1 h. Concentrations for the antibody standard were 0 μM, 50 μM, 75 μM, 100 μM, 150 μM, 200 μM, 250 μM, and 300 μM. After incubation 3x wash and one rinse were performed and 150μl/well detection antibody, anti-mouse IgG horseradish peroxidase conjugate at 0.4μg/ml (Pierce Protein Products, USA) was added for incubation of 1 h. Six washes and one rinse repeated followed by addition of 150μl ABTS substrate added for 20 min incubation. 100μl stop solution was added (1% Sodium dodecyl sulfate) and final absorbance measured at 410nm in BioTek Synergy2 plate reader.

### Statistical analysis

Statistical analysis was performed utilizing the Sigmaplot 12.3 software package by Systat Software Inc. All data are presented as mean ± SD. In all studies, differences between groups were considered significant at *P* < 0.05.

A student’s two tailed unpaired t-test was used to determine *in vitro* trans-cinnamate production in control vs. pHENOMMenal cell lines using triplicate wells for each condition.

Preliminary animal studies, at two animals per experimental repeat with three repeats of the experiment, comparing the standard 5LL2 chow to 5LL2 chow with treatment probiotic were pooled to enable the use of a one-tailed paired t-test for comparison of pre and post treatment values. All samples for the seventh day of treatment experimental time point were pooled and tested for significance using a one tailed t-test with n = 12. An example of a single experimental run is available in S1 Fig for comparative purposes.

Animals producing samples that were not lost due to equipment failure were analyzed at pre treatment, seven days and fourteen days of treatment time points. Again, a one-tailed paired t-test was used to analyze the data between treatment time points and pre treatment values, and to compare the two treatment time points with each other. To test for anti-AvPAL antibodies, blood samples during and after probiotic treatment were compared to baseline samples (prior to treatment) for each individual mouse due to the large variance in baseline values, and as such there was no statistical analysis. Based on data from positive control mice, and a typical exponential increase when the immune system responds to an immunogenic peptide, a 5x increase in anti-AvPAL antibodies above baseline (pretreatment) was used to indicate a systemic immune response to the AvPAL in the probiotic treatment.

## Results

### *In vitro*—AvPAL activity

**Fig 1:**
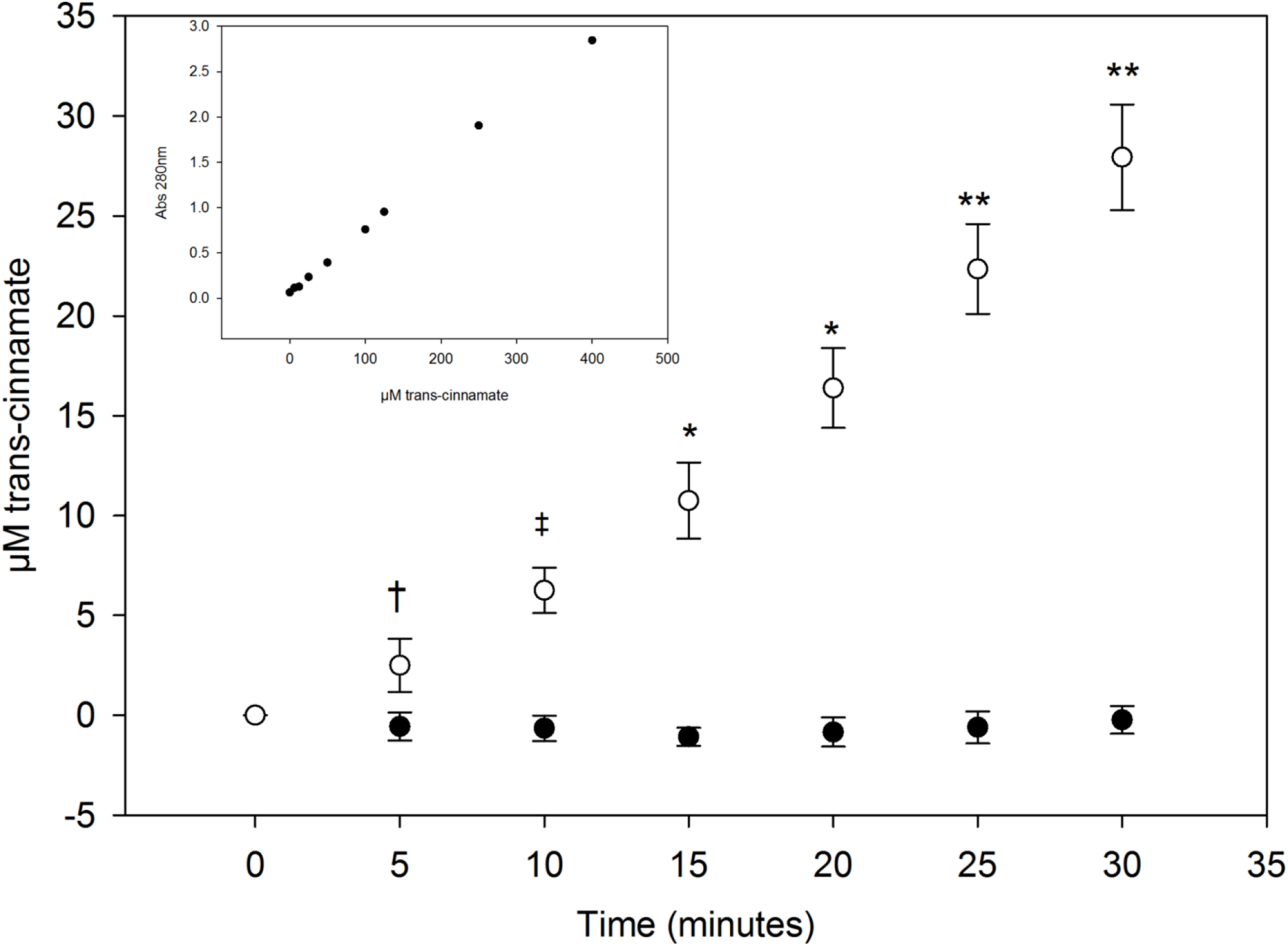
Trans-cinnamate production by cell line. Trans-cinnamate produced as a result of phe catabolism by vector control (pSLERGT) or pHENOMMenal (pHENOMM) cell lysates when held at 37°C. Time points 5, 10, 15, 20, 25, and 30 minutes showed significant production of trans-cinnamate in pHENOMMenal cell lysate compared to the control pSLERGT lysate (*P* < 0.05, 0.001, 0.0005, 0.001, 0.00025, and 0.0001 respectively). Each data point represents the mean of triplicate wells at 5 minute intervals starting at 0 minutes and ending at 30 minutes. Error bars indicate standard deviation. (Trans-cinnamate standard curve inset).

PAL enzymes produce one molecule of trans-cinnamate for every molecule of phe catabolized and production of trans-cinnamate increases absorbance of 280nm light, enabling spectrophotometric detection of AvPAL activity *in vitro*[21]. Lysate from control cells caused no detectable increase in trans-cinnamate while lysate from pHENOMMenal cells produced a significant increase in absorbance at 280nm (*P* < 0.05, 0.001, 0.0005, 0.001, 0.00025, and 0.0001 for time points of 5, 10, 15,20, 25, and 30 minutes respectively), indicating *in vitro* efficacy of the AvPAL enzyme in pHENOMMenal (Fig 1)

### Short term probiotic feeding

**Fig 2:**
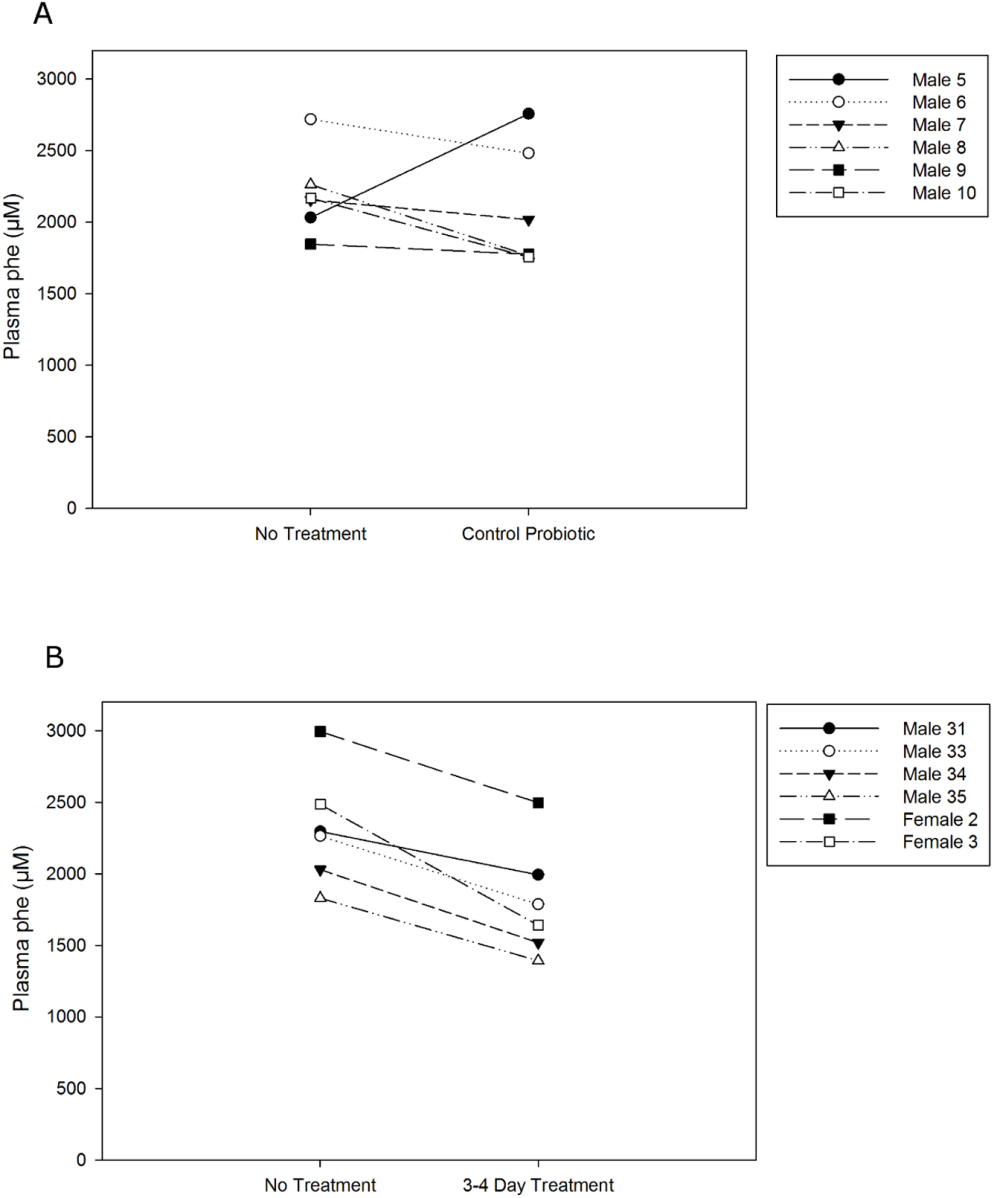
Preliminary efficacy of ingested probiotics. Blood samples were collected from PKU mice prior to treatment, and samples were collected from the same animals after treatment. Each dot and tie line represents one animal. (A) PKU male mice were fed control probiotic for four days prior to second sample collection. Data shown are results pooled from two independent experimental runs. *P* > 0.25. (B) PKU mice were fed pHENOMMenal probiotic for four days (males, circles) or three days (females, triangles) prior to second sample collection. Data shown are pooled results from three independent experimental runs, *P* < 0.0005.

Preliminary three to four day feeding experiments were conducted to determine *in vivo* attributes of pHENOMMenal and control probiotic feeding using the mouse model of PKU. Cells for these experiments were harvested for freeze drying during exponential growth to be consistent with the previously collected *in vitro* enzymatic data. Mice ingesting control probiotic for a period of four days did not demonstrate a reduction in plasma phe values (Fig 2A, *P* > 0.25). Conversely, ingesting pHENOMMenal probiotic for three to four days did significantly reduce plasma phe values (Fig 2B, mean reduction of 511.6 ± 179.3 μM phe, *P* < 0.0005)

Prior to probiotic treatment feces from the mouse colony were incapable of growing bacterial colonies on Lactobacillus selective/preferential MRS agar plates with 5μg/ml of erythromycin (ery) in anaerobic conditions at 37°C. However colonies were detected in the same culture conditions from feces collected 24 hours after the start of feeding with growth phase harvested pHENOMMenal, an ery resistant probiotic, indicating survival of the engineered Lactobacillus in the mouse gastrointestinal tract. Preliminary feeding of probiotic for four days failed to produce permanent colonization by the probiotic (as demonstrated by a lack of ery resistant colonies from feces collected 2 days after cessation of probiotic feeding.)

### Blood phe reduction by pHENOMMenal extended feeding studies

**Fig 3:**
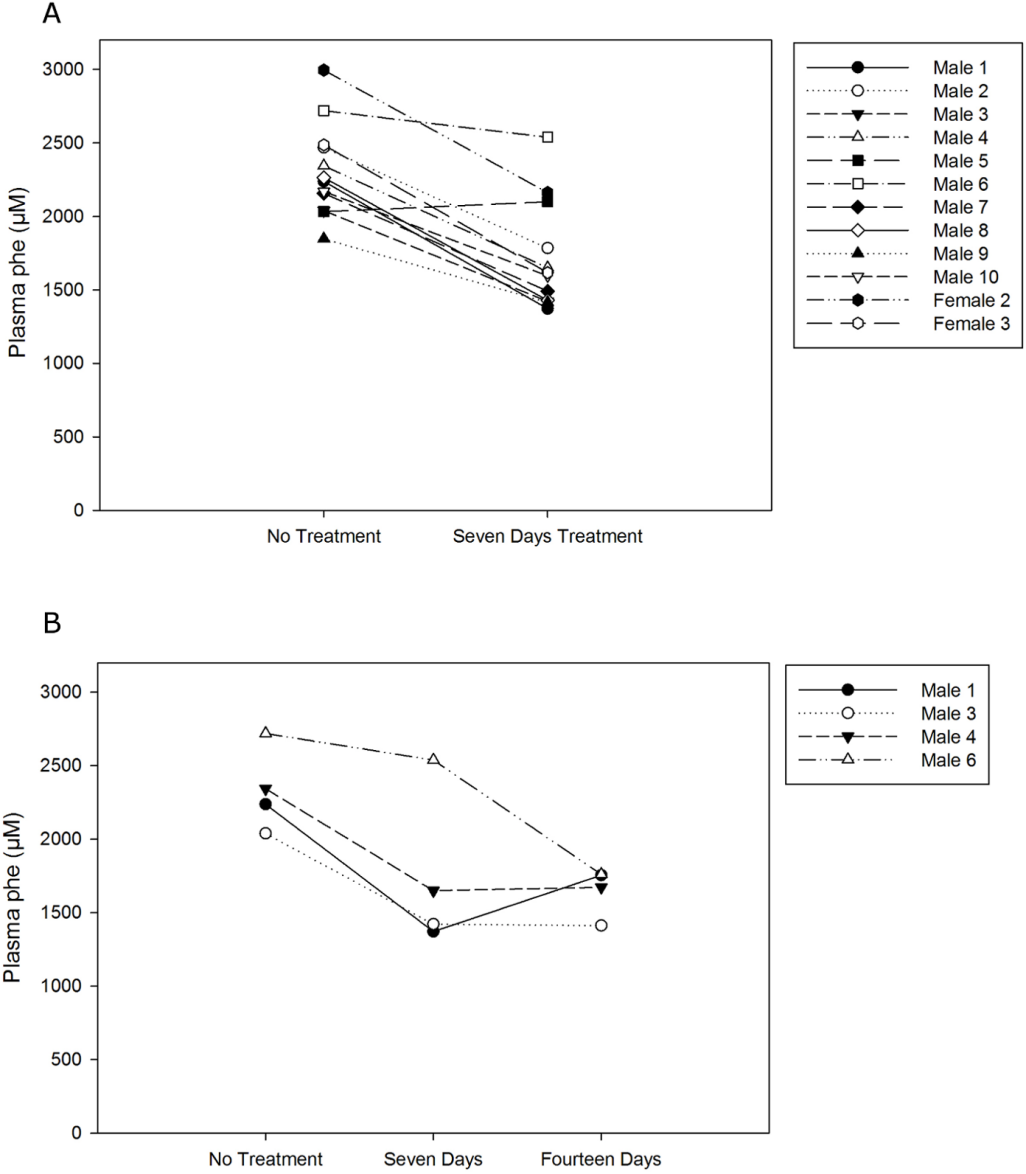
Effect of seven day pHENOMMenal treatment on blood phe levels. (A) Blood was collected prior to and after PKU mice were fed pHENOMMenal probiotic for seven days. Each dot connected by a line indicates an individual mouse (*P* < 1.0^−5^, n = 12). Data is a pool of four independent experiments, three experiments used male animals and one experiment used female animals. (B) Blood was collected prior to treatment, after seven days of treatment, and again after fourteen days of treatment with pHENOMMenal probiotic. Significant reduction in blood phe compared to the control is observed at seven days and fourteen days (*P* < 0.025 and *P* < 0.005 respectively), but no significant reduction is observed between day seven and day fourteen (*P* > 0.25). Data representative of four animals (n = 4), numbers correspond to the same animal in the graph for 3A.

**Fig 4:**
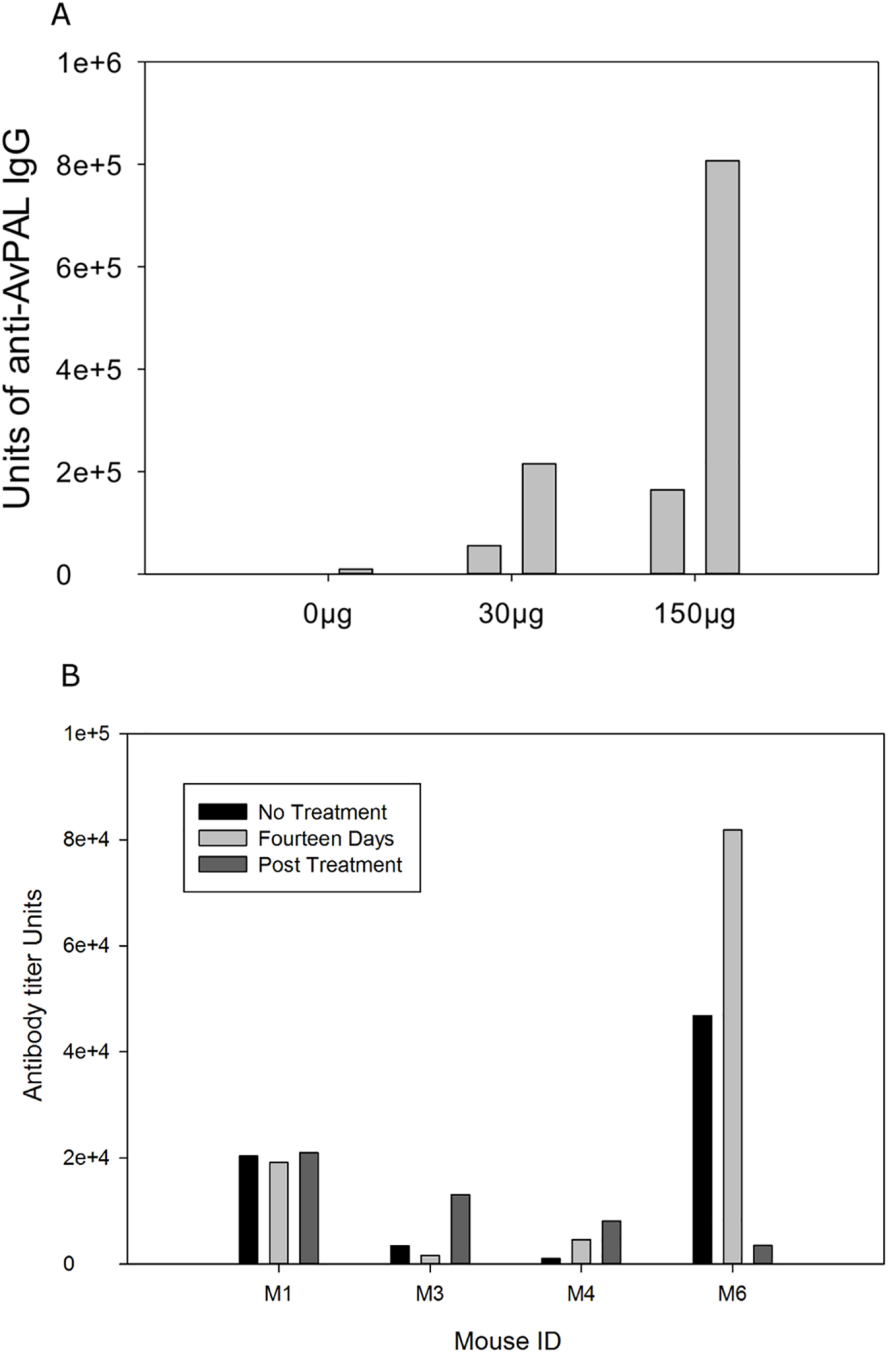
Anti-AvPAL antibody titers. (A) Anti-AvPAL IgG titers from animals injected on days zero, seven, and fourteen with 0μg, 30μg, or 150μg AvPAL. (B) anti-AvPAL IgG titers from each mouse in Fig 4 throughout and after the fourteen day treatment study. Collections shown from left to right for each animal are prior to treatment, after fourteen days of pHENOMMenal treatment, and four months after cessation of treatment

A seven day feeding experiment was conducted to further determine efficacy of pHENOMMenal probiotic treatment. Plasma phe was significantly reduced in the seven day feeding by a mean of 598.6 ± 289.5 μM (Fig 3A, *P* < 1.0 x 10^−5^, n = 12). A small cohort of animals continued pHENOMMenal probiotic treatment for fourteen days. Animals maintained a similarly reduced blood phe value after fourteen days of treatment as compared to their seven day treatment values (Fig 4, *P* > 0.25, n = 4). An independent experimental run is shown in S1 Fig with statistical information for reference.

To determine possible long term colonization of the PKU mouse gut by pHENOMMenal when ingestion of the probiotic occurred for more than 4 days, fecal samples were examined for microbial growth characteristics four months after cessation of probiotic feeding. Cells grew in the *L*. *reuteri* 100–23 + pHENOMM specific conditions (MRS broth + ery), but not under the *E*. *coli* + pHENOMM specific conditions (LB + amp). At eight months post treatment, fecal samples produced no growth consistent with pHENOMMenal.

### Anti-AvPAL IgGs

Due to AvPAL's immunogenic properties and the potential for a systemic immune response measurable via immunoglobulin production, anti-AvPAL IgG ELISA were conducted on plasma from the fourteen day pHENOMMenal treatments. Animals never treated with pHENOMMenal were used as controls for the assay. The negative control mouse received injections with 0μg AvPAL and produced a background of anti-AvPAL antibody titer of 9,000 units (Fig 4A). Positive controls of 30 μg AvPAL/week and 150μg AvPAL/week produced significant antibody titers on day fourteen (56,000 units and 191,000 units respectively), and this titer increased by day 21 to 201,000 units and 807,000 units respectively (Fig 4A). The observed value of a 5-fold increase in antibody titer in the 30 μg positive control fourteen days post first injection became the threshold to indicate the occurrence of a systemic immune response to AvPAL.

Blood plasma collected prior to administration of probiotic served as a baseline of Anti-AvPAL for each animal. Plasma anti-AvPAL IgG antibodies were quantified using a standard curve (S2 Fig). Anti-AvPAL IgG antibody titers failed to rise above the derived significance threshold of a 5-fold increase in all time points examined (Fig 4).

## Discussion

Results from the present study support a potential new therapy for PKU disease, by metabolizing phe in the gut of a PKU model mouse and showing a reduction in resultant phe levels in the blood.

As expected from the results of the *in vitro* studies, feeding PKU mice control probiotic did not result in a reduction in plasma phe concentrations. In addition, a threshold of therapeutic function was not observed when pHENOMMenal was administered in reduced phe chow. This indicated the ability of the engineered microbe to reduce blood phe in PKU animals regardless of dietary phe intake for the diets tested. Freeze dried pHENOMMenal cells harvested in stationary phase were unable to reduce plasma phe despite retaining viability (cfu count) and functional AvPAL, indicating a discrepancy in *L*. *reuteri* 100–23's tolerance of acid/bile, which is dependent on the growth phase of harvested cells.

Naked PAL enzymes were highly immunogenic when injected into mice and humans [19], and this immune response eliminates efficacy of therapy over time [18]. Confinement of AvPAL to the gut should prevent systemic immunodetection of the enzyme by the same immunological ignorance afforded to resident gut microbes [20]. This assumption was supported by data in our animal feeding studies by a lack of AvPAL specific IgG antibodies in the blood. Lack of systemic antibodies and continued efficacy of treatment indicated AvPAL enzyme is likely performing phe catabolism while remaining in the gut of the PKU mouse. Previously described rapid cleavage of AvPAL by intestinal proteases [9] and results from our *in vitro* studies lead us to conclude that not only is AvPAL performing phe catabolism in the gut, but AvPAL is remaining within the pHENOMMenal cells when *in vivo*.

Previous research indicated *L*. *reuteri* 100–23 was capable of permanent colonization of the mouse intestine when animals lacked *Lactobacilli* [14]. Post treatment fecal samples demonstrated growth consistent with erythromycin resistant *L*. *reuteri* 100–23 in animals receiving seven or more days of pHENOMMenal, but not in animals fed pHENOMMenal for four days. This indicated that more than 4 days of oral pHENOMMenal administration in PKU animals was necessary for successful long term colonization of the conventional mouse gut. As demonstrated by the lack of cultivable microbes at eight months post treatment, permanent colonization of the mouse gut by pHENOMMenal does not appear to occur. Although this organism seemed to be capable of colonizing the PKU mouse gut, the ability of pHENOMMenal to colonize the healthy mouse gut has not been assessed.

None of our studies resulted in plasma phe concentrations of a healthy mouse (138±12μM, [27]). Higher cfu counts, the use of other bacteria, additional/increased phe uptake by the bacterium, and increased AvPAL gene copy number may improve the function of this therapy. These results may also indicate a genetically engineered microbe such as pHENOMMenal cannot completely alleviate the need for a reduced phe diet. Many differences in digestive systems of a mouse and human made attempting to find the ideal dosage for treatment of PKU in the mouse model inadvisable as the data may or may not translate into comparable human efficacy. However, even if this therapy is only able to supplement the PKU diet, many PKU patients and parents of PKU patients have expressed the desire to use this therapy even if it only allows for a 10–20% increase in daily phe ingestion (pers. com. NPKUA 2014). pHENOMMenal probiotic would be taken orally, likely on a daily basis. This is preferred to daily injections(such as PEG-PAL) by eliminating the need for injection, and the noted prevalence of immunological reactions to the injection of PAL and PEG- PAL molecules [9, 18, 19].

Microbial phe catabolism may be increased by expressing AvPAL in the bacteria of the large intestine, especially in a secreted form. Although amino acid absorption occurs in the small intestine, it is possible AvPAL in the large intestine would create a phe sink and draw blood phe into the large intestine for catabolism into trans-cinnamate and ammonia. Secretion of AvPAL in the large intestine would ensure AvPAL has direct access to free (non-peptide) phe, and secretion of AvPAL would reduce the metabolic burden the enzyme might otherwise place on the microbe. Secretion of AvPAL in the small intestine is also a consideration, though digestive enzymes at this location would cleave AvPAL rapidly [9].

Several differences exist between the mouse model of PKU and human patients with the disease. Mice eat constantly, and a mouse on standard chow (5LL2 chow) ingests 240mg phe/kg body weight daily while the average human ingests 70mg phe/kg body weight (pers.com. NPKUA). Perhaps as a result of the heightened phe ingestion, plasma phe in healthy and PKU mice is higher (138±12μM, [27] and 2268μM,data from our colony respectively) than in healthy and PKU humans (60 μM and 1200 μM, respectively pers.com. NPKUA). The mouse gastrointestinal tract is optimized to handle the increased caloric load (cal/kg body weight) and gastrointestinal transit time is reported as 10 hours [22]. Gastrointestinal transit times in the human body are variable, with dye tests indicating complete passage starting at 12 hours with dye excretion continuing for up to 48 hours, although times greater than 72 hours are also observed [28]. Given these differences it is unknown how they will alter efficacy of pHENOMMenal treatment when translating this new therapy into human trials.

Permanent colonization of the human intestine by an engineered microbe is unlikely to pass FDA approval, especially if the genetically modified microbe can colonize *healthy* human intestines. Prior to this study, *L*. *reuteri* 100–23 demonstrated the ability to permanently colonize the small intestine under certain conditions [14]. To reduce the possibility of permanent colonization of a human variant, strains with little to no permanent colonization of the human intestine will be considered for potential clinical use. Similarly, plasmid based techniques from this study are not ideal for human use. A chromosomal gene insertion is more likely to be accepted by the FDA for a human version of this therapy.

In summary, the genetically modified probiotic referred to as pHENOMMenal in the present study, was able to significantly reduce plasma phe in the mouse model of PKU. These results provide evidence that a microbe may be genetically engineered to treat a eukaryotic host's metabolic defects. pHENOMMEnal may become a model for an inexpensive treatment for PKU and creation of a human version of this probiotic therapy is currently underway.

## Supporting information

S1 File DNA sequences of synthesized genes and plasmids used in this work. (PDF)

S1 Fig Single experimental run of seven day treatment. Four male animals were used for this experimental run with blood collected pre treatment and after seven days of treatment with pHENOMMenal probiotic. Mean plasma phe decrease was 715.1 ± 106.5μM, *P* < 0.0005.
(PDF)

S2 Fig Standard curve for anti-AvPAL antibody assay. (TIF)
