# Supplementary material for: Genetically engineered probiotic for the treatment of phenylketonuria (PKU); assessment of a novel treatment in vitro and in the PAHenu2 mouse model of PKU
Source: PLoS One. 2017 May 17;12(5):e0176286. doi: 10.1371/journal.pone.0176286 (PMC5435137; doi:10.1371/journal.pone.0176286)
Supplement: S1 File — (PDF) [file pone.0176286.s001.pdf]

FuzErmAvPAL, forward orientation

```
   1 GCATGCTAGC TATAGTTCTA GAGGATACCT GGTTGATTAA CGTTAGCCTG GCTACGTATA
  61 CTCCTGGAAG TATTAATAGA CGACCTAGGA TGCATATGTT CAAGAGTGTG TTGATAGTGC
 121 AGTATCTTAA AATTTTGTAT AATAGGAATT GAAGTTAAAT TAGATGCTAA AAATTTGTAA
 181 TTAAGAAGGA GTAGGTATAA ATGAAGACTT TATCTCAAGC ACAATCTAAG ACTAGTTCTC
 241 AACAATTCTC ATTTACTGGA AACTCAAGTG CTAATGTTAT CATTGGTAAT CAAAAGCTTA
 301 CTATTAACGA TGTTGCACGG GTTGCACGCA ATGGTACTTT AGTTAGTTTA ACTAACAACA
 361 CTGACATTTT ACAAGGCATT CAAGCTAGTT GCGACTATAT CAATAACGCT GTTGAATCAG
 421 GAGAACCAAT TTATGGTGTT ACTTCAGGAT TCGGTGGAAT GGCAAACGTC GCTATTAGTC
 481 GTGAACAAGC TTCTGAACTT CAAACTAATC TTGTTTGGTT TCTTAAAACA GGAGCTGGTA
 541 ATAAACTTCC ATTAGCAGAT GTTCGTGCTG CTATGTTATT ACGTGCAAAT TCACACATGC
 601 GTGGCGCTTC TGGTATTCGT CTTGAATTAA TTAAGCGTAT GGAAATTTTC CTTAACGCCG
 661 GTGTTACACC TTATGTTTAC GAATTCGGAT CAATTGGTGC AAGTGGAGAT CTTGTTCCTT
 721 TATCTTATAT TACAGGTTCT TTAATTGGTT TGGATCCATC ATTCAAAGTT GATTTTAATG
 781 GTAAAGAAAT GGATGCCCCA ACAGCTCTTC GACAATTAAA TTTATCACCA CTTACATTAC
 841 TTCCAAAAGA AGGTCTTGCT ATGATGAATG GTACATCAGT TATGACGGGA ATTGCTGCTA
 901 ATTGCGTTTA TGATACTCAA ATTCTTACTG CTATTGCCAT GGGTGTTCAT GCCTTAGATA
 961 TTCAAGCACT TAATGGCACA AATCAAAGTT TCCACCCATT CATTCACAAT TCTAAACCTC
1021 ACCCAGGTCA ACTTTGGGCT GCTGATCAAA TGATTTCTCT TTTAGCTAAT AGTCAACTTG
1081 TTCGCGACGA ATTAGATGGT AAGCATGATT ATCGTGATCA TGAACTTATT CAAGACCGCT
1141 ATAGTTTACG TTGCTTACCA CAATATTTGG GTCCTATTGT TGATGGAATT TCACAAATTG
1201 CTAAGCAAAT TGAAATCGAA ATTAATAGTG TCACTGACAA TCCATTAATT GACGTTGATA
1261 ACCAAGCCTC ATACCATGGC GGTAACTTTT TGGGTCAATA TGTTGGCATG GGCATGGACC
1321 ACTTACGTTA CTACATTGGT CTTCTTGCAA AGCATTTGGA TGTCCAAATT GCACTTTTGG
1381 CCAGTCCAGA ATTCAGTAAC GGACTTCCAC CATCATTATT GGGAAACCGG GAACGAAAGG
1441 TAAACATGGG TCTTAAGGGT TTGCAAATTT GTGGCAACTC AATTATGCCA TTGTTGACTT
1501 TTTACGGAAA TTCAATTGCT GATCGCTTTC CTACACACGC CGAACAATTT AACCAAAATA
1561 TTAACTCTCA AGGATACACT TCTGCTACGT TAGCACGACG GTCTGTTGAT ATTTTTCAAA
1621 ACTACGTAGC TATTGCATTA ATGTTTGGAG TACAAGCTGT TGATTTACGG ACTTACAAAA
1681 AGACGGGCCA TTATGATGCT CGTGCTTGTT TAAGTCCAGC TACAGAACGT CTTTATTCAG
1741 CAGTACGTCA TGTTGTCGGC CAAAAACCTA CATCAGATCG GCCATATATT TGGAACGATA
1801 ACGAACAAGG ACTTGACGAA CATATTGCTC GTATTTCTGC TGATATCGCT GCTGGTGGTG
1861 TAATTGTTCA AGCCGTACAA GATATCCTTC CTTGTTTACA CTAATAATGA TAAGTTAAGG
1921 GGTGCATAAA CTGCATCCCT TAACTTATCA AAAAAAAGTC GAC
```

pSLER1

1 TCTTCCGCTT CCTCGCTCAC TGACTCGCTG CGCTCGGTCG TTCGGCTGCG GCGAGCGGTA
61 TCAGCTCACT CAAAGGCGGT AATACGGTTA TCCACAGAAT CAGGGGATAA CGCAGGAAAG
121 AACATGTGAG CAAAAGGCCA GCAAAAGGCC AGGAACCGTA AAAAGGCCGC GTTGCTGGCG
181 TTTTTCCATA GGCTCCGCCC CCCTGACGAG CATCACAAAA ATCGACGCTC AAGTCAGAGG
241 TGGCGAAACC CGACAGGACT ATAAAGATAC CAGGCGTTTC CCCCTGGAAG CTCCCTCGTG
301 CGCTCTCCTG TTCCGACCCT GCCGCTTACC GGATACCTGT CCGCCTTTCT CCCTTCGGGA
361 AGCGTGGCGC TTTCTCATAG CTCACGCTGT AGGTATCTCA GTTCGGTGTA GGTCGTTCGC
421 TCCAAGCTGG GCTGTGTGCA CGAACCCCCC GTTCAGCCCG ACCGCTGCGC CTTATCCGGT
481 AACTATCGTC TTGAGTCCAA CCCGGTAAGA CACGACTTAT CGCCACTGGC AGCAGCCACT
541 GGTAACAGGA TTAGCAGAGC GAGGTATGTA GGCGGTGCTA CAGAGTTCTT GAAGTGGTGG
601 CCTAACTACG GCTACACTAG AAGAACAGTA TTTGGTATCT GCGCTCTGCT GAAGCCAGTT
661 ACCTTCGGAA AAAGAGTTGG TAGCTCTTGA TCCGGCAAAC AAACCACCGC TGGTAGCGGT
721 GGTTTTTTTG TTTGCAAGCA GCAGATTACG CGCAGAAAAA AAGGATCTCA AGAAGATCCT
781 TTGATCTTTT CTACGGGGTC TGACGCTCAG TGGAACGAAA ACTCACGTTA AGGGATTTTG
841 GTCATGAGAT TATCAAAAAG GATCTTCACC TAGATCCTTT TAAATTAAAA ATGAAGTTTT
901 AAATCAATCT AAAGTATATA TGAGTAAACT TGGTCTGACA GTTACCAATG CTTAATCAGT
961 GAGGCACCTA TCTCAGCGAT CTGTCTATTT CGTTCATCCA TAGTTGCCTG ACTCCCCGTC
1021 GTGTAGATAA CTACGATACG GGAGGGCTTA CCATCTGGCC CCAGTGCTGC AATGATACCG
1081 CGAGACCCAC GCTCACCGGC TCCAGATTTA TCAGCAATAA ACCAGCCAGC CGGAAGGGCC
1141 GAGCGCAGAA GTGGTCCTGC AACTTTATCC GCCTCCATCC AGTCTATTAA TTGTTGCCGG
1201 GAAGCTAGAG TAAGTAGTTC GCCAGTTAAT AGTTTGCGCA ACGTTGTTGC CATTGCTACA
1261 GGCATCGTGG TGTCACGCTC GTCGTTTGGT ATGGCTTCAT TCAGCTCCGG TTCCCAACGA
1321 TCAAGGCGAG TTACATGATC CCCCATGTTG TGCAAAAAAG CGGTTAGCTC CTTCGGTCCT
1381 CCGATCGTTG TCAGAAGTAA GTTGGCCGCA GTGTTATCAC TCATGGTTAT GGCAGCACTG
1441 CATAATTCTC TTACTGTCAT GCCATCCGTA AGATGCTTTT CTGTGACTGG TGAGTACTCA
1501 ACCAAGTCAT TCTGAGAATA GTGTATGCGG CGACCGAGTT GCTCTTGCCC GGCGTCAATA
1561 CGGGATAATA CCGCGCCACA TAGCAGAACT TTAAAAGTGC TCATCATTGG AAAACGTTCT
1621 TCGGGGCGAA AACTCTCAAG GATCTTACCG CTGTTGAGAT CCAGTTCGAT GTAACCCACT
1681 CGTGCACCCA ACTGATCTTC AGCATCTTTT ACTTTCACCA GCGTTTCTGG GTGAGCAAAA
1741 ACAGGAAGGC AAAATGCCGC AAAAAAGGGA ATAAGGGCGA CACGGAAATG TTGAATACTC
1801 ATACTCTTCC TTTTTCAATA TTATTGAAGC ATTTATCAGG GTTATTGTCT CATGAGCGGA
1861 TACATATTTG AATGTATTTA GAAAAATAAA CAAATAGGGG TTCCGCGCAC ATTTCCCCGA
1921 AAAGTGCCAC CTGACGTCTA AGAAACCATT ATTATCATGA CATTAACCTA TAAAAATAGG
1981 CGTATCACGA GGCCCTTTCG TCTCGCGCGT TTCGGTGATG ACGGTGAAAA CCTCTGACAC
2041 ATGCAGCTCC CGGAGACGGT CACAGCTTGT CTGTAAGCGG ATGCCGGGAG CAGACAAGCC
2101 CGTCAGGGCG CGTCAGCGGG TGTTGGCGGG TGTCGGGGCT GGCTTAACTA TGCGGCATCA
2161 GAGCAGATTG TACTGAGAGT GCACCATAAA ATTGTAAACG TTAATATTTT GTTAAAATTC
2221 GCGTTAAATT TTTGTTAAAT CAGCTCATTT TTTAACCAAT AGGCCGAAAT CGGCAAAATC
2281 CCTTATAAAT CAAAAGAATA GCCCGAGATA GGGTTGAGTG TTGTTCCAGT TTGGAACAAG
2341 AGTCCACTAT TAAAGAACGT GGACTCCAAC GTCAAAGGGC GAAAAACCGT CTATCAGGGC
2401 GATGGCCCAC TACGTGAACC ATCACCCAAA TCAAGTTTTT TGGGGTCGAG GTGCCGTAAA
2461 GCACTAAATC GGAACCCTAA AGGGAGCCCC CGATTTAGAG CTTGACGGGG AAAGCCGGCG
2521 AACGTGGCGA GAAAGGAAGG GAAGAAAGCG AAAGGAGCGG GCGCTAGGGC GCTGGCAAGT
2581 GTAGCGGTCA CGCTGCGCGT AACCACCACA CCCGCCGCGC TTAATGCGCC GCTACAGGGC
2641 GCGTACTATG GTTGCTTTGA CGTATGCGGT GTGAAATACC GCACAGATGC GTAAGGAGAA
2701 AATACCGCAT CAGGCGCCAT TCGCCATTCA GGCTGCGCAA CTGTTGGGAA GGGCGATCGG
2761 TGCGGGCCTC TTCGCTATTA CGCCAGCTGG CGAAAGGGGG ATGTGCTGCA AGGCGATTAA
2821 GTTGGGTAAC GCCAGGGTTT TCCCAGTCAC GACGTTGTAA AACGACGGCC AGTGAATTCG
2881 AATGGCCATG GGACGTCGAC CTGAGGTAAT TATAACCCGG GCCCTATATA TGGATCCAAT
2941 TGCAATGATC ATCATGACAG ATCTGCGCGC GATCGATATC AGCGCTTTAA ATTTGCGCAT
3001 GCTAGCTATA GTTCTAGAGG TACCGGTTGT TAACGTTAGC CGGCTACGTA TACTCCGGAA
3061 TATTAATAGG CCTAGGATGC ATATGTTAAG AGTGTGTTGA TAGTGCAGTA TCTTAAAATT
3121 TTGTATAATA GGAATTGAAG TTAAATTAGA TGCTAAAAAT TTGTAATTAA GAAGGAGTGA
3181 TTACATGAAC AAAAATATAA AATATTCTCA AAACTTTTTA ACGAGTGAAA AAGTACTCAA
3241 CCAAATAATA AAACAATTGA ATTTAAAAGA AACCGATACC GTTTACGAAA TTGGAACAGG
3301 TAAAGGGCAT TTAACGACGA AACTGGCTAA AATAAGTAAA CAGGTAACGT CTATTGAATT

3361 AGACAGTCAT CTATTCAACT TATCGTCAGA AAAATTAAAA CTGAATACTC GTGTCACTTT
3421 AATTCACCAA GATATTCTAC AGTTTCAATT CCCTAACAAA CAGAGGTATA AAATTGTTGG
3481 GAGTATTCCT TACCATTTAA GCACACAAAT TATTAAAAAA GTGGTTTTTG AAAGCCATGC
3541 GTCTGACATC TATCTGATTG TTGAAGAAGG ATTCTACAAG CGTACCTTGG ATATTCACCG
3601 AACACTAGGG TTGCTCTTGC ACACTCAAGT CTCGATTCAG CAATTGCTTA AGCTGCCAGC
3661 GGAATGCTTT CATCCTAAAC CAAAAGTAAA CAGTGTCTTA ATAAAACTTA CCCGCCATAC
3721 CACAGATGTT CCAGATAAAT ATTGGAAGCT ATATACGTAC TTTGTTTCAA AATGGGTCAA
3781 TCGAGAATAT CGTCAACTGT TTACTAAAAA TCAGTTTCAT CAAGCAATGA AACACGCCAA
3841 AGTAAACAAT TTAAGTACCG TTACTTATGA GCAAGTATTG TCTATTTTTA ATAGTTATCT
3901 ATTATTTAAC GGGAGGAAAT AATTCTATGA GTCGCTTTTG TAAATTTGGA AAGTTACACG
3961 TTACTAAAGG GAATGTAGAT AAATTATTAG GTATACTACT GACAGCTTCC AAGCATATGG
4021 CGGCCGCCTG CAGCTGGCGC CATCGATACG CGTACGTCGC GACCGCGGAC ATGTACAGAG
4081 CTCGAGAAGT ACTAGTGGCC ACGTGGGCCG TGCACCTTAA GCTTGGCGTA ATCATGGTCA
4141 TAGCTGTTTC CTGTGTGAAA TTGTTATCCG CTCACAATTC CACACAACAT ACGAGCCGGA
4201 AGCATAAAGT GTAAAGCCTG GGGTGCCTAA TGAGTGAGCT AACTCACATT AATTGCGTTG
4261 CGCTCACTGC CCGCTTTCCA GTCGGGAAAC CTGTCGTGCC AGCTGCATTA ATGAATCGGC
4321 CAACGCGCGG GGAGAGGCGG TTTGCGTATT GGGCGC

pSLERGT

1 TCTTCCGCTT CCTCGCTCAC TGACTCGCTG CGCTCGGTCG TTCGGCTGCG GCGAGCGGTA
61 TCAGCTCACT CAAAGGCGGT AATACGGTTA TCCACAGAAT CAGGGGATAA CGCAGGAAAG
121 AACATGTGAG CAAAAGGCCA GCAAAAGGCC AGGAACCGTA AAAAGGCCGC GTTGCTGGCG
181 TTTTTCCATA GGCTCCGCCC CCCTGACGAG CATCACAAAA ATCGACGCTC AAGTCAGAGG
241 TGGCGAAACC CGACAGGACT ATAAAGATAC CAGGCGTTTC CCCCTGGAAG CTCCCTCGTG
301 CGCTCTCCTG TTCCGACCCT GCCGCTTACC GGATACCTGT CCGCCTTTCT CCCTTCGGGA
361 AGCGTGGCGC TTTCTCATAG CTCACGCTGT AGGTATCTCA GTTCGGTGTA GGTCGTTCGC
421 TCCAAGCTGG GCTGTGTGCA CGAACCCCCC GTTCAGCCCG ACCGCTGCGC CTTATCCGGT
481 AACTATCGTC TTGAGTCCAA CCCGGTAAGA CACGACTTAT CGCCACTGGC AGCAGCCACT
541 GGTAACAGGA TTAGCAGAGC GAGGTATGTA GGCGGTGCTA CAGAGTTCTT GAAGTGGTGG
601 CCTAACTACG GCTACACTAG AAGAACAGTA TTTGGTATCT GCGCTCTGCT GAAGCCAGTT
661 ACCTTCGGAA AAAGAGTTGG TAGCTCTTGA TCCGGCAAAC AAACCACCGC TGGTAGCGGT
721 GGTTTTTTTG TTTGCAAGCA GCAGATTACG CGCAGAAAAA AAGGATCTCA AGAAGATCCT
781 TTGATCTTTT CTACGGGGTC TGACGCTCAG TGGAACGAAA ACTCACGTTA AGGGATTTTG
841 GTCATGAGAT TATCAAAAAG GATCTTCACC TAGATCCTTT TAAATTAAAA ATGAAGTTTT
901 AAATCAATCT AAAGTATATA TGAGTAAACT TGGTCTGACA GTTACCAATG CTTAATCAGT
961 GAGGCACCTA TCTCAGCGAT CTGTCTATTT CGTTCATCCA TAGTTGCCTG ACTCCCCGTC
1021 GTGTAGATAA CTACGATACG GGAGGGCTTA CCATCTGGCC CCAGTGCTGC AATGATACCG
1081 CGAGACCCAC GCTCACCGGC TCCAGATTTA TCAGCAATAA ACCAGCCAGC CGGAAGGGCC
1141 GAGCGCAGAA GTGGTCCTGC AACTTTATCC GCCTCCATCC AGTCTATTAA TTGTTGCCGG
1201 GAAGCTAGAG TAAGTAGTTC GCCAGTTAAT AGTTTGCGCA ACGTTGTTGC CATTGCTACA
1261 GGCATCGTGG TGTCACGCTC GTCGTTTGGT ATGGCTTCAT TCAGCTCCGG TTCCCAACGA
1321 TCAAGGCGAG TTACATGATC CCCCATGTTG TGCAAAAAAG CGGTTAGCTC CTTCGGTCCT
1381 CCGATCGTTG TCAGAAGTAA GTTGGCCGCA GTGTTATCAC TCATGGTTAT GGCAGCACTG
1441 CATAATTCTC TTACTGTCAT GCCATCCGTA AGATGCTTTT CTGTGACTGG TGAGTACTCA
1501 ACCAAGTCAT TCTGAGAATA GTGTATGCGG CGACCGAGTT GCTCTTGCCC GGCGTCAATA
1561 CGGGATAATA CCGCGCCACA TAGCAGAACT TTAAAAGTGC TCATCATTGG AAAACGTTCT
1621 TCGGGGCGAA AACTCTCAAG GATCTTACCG CTGTTGAGAT CCAGTTCGAT GTAACCCACT
1681 CGTGCACCCA ACTGATCTTC AGCATCTTTT ACTTTCACCA GCGTTTCTGG GTGAGCAAAA
1741 ACAGGAAGGC AAAATGCCGC AAAAAAGGGA ATAAGGGCGA CACGGAAATG TTGAATACTC
1801 ATACTCTTCC TTTTTCAATA TTATTGAAGC ATTTATCAGG GTTATTGTCT CATGAGCGGA
1861 TACATATTTG AATGTATTTA GAAAAATAAA CAAATAGGGG TTCCGCGCAC ATTTCCCCGA

1921 AAAGTGCCAC CTGACGTCTA AGAAACCATT ATTATCATGA CATTAACCTA TAAAAATAGG
1981 CGTATCACGA GGCCCTTTCG TCTCGCGCGT TTCGGTGATG ACGGTGAAAA CCTCTGACAC
2041 ATGCAGCTCC CGGAGACGGT CACAGCTTGT CTGTAAGCGG ATGCCGGGAG CAGACAAGCC
2101 CGTCAGGGCG CGTCAGCGGG TGTTGGCGGG TGTCGGGGCT GGCTTAACTA TGCGGCATCA
2161 GAGCAGATTG TACTGAGAGT GCACCATAAA ATTGTAAACG TTAATATTTT GTTAAAATTC
2221 GCGTTAAATT TTTGTTAAAT CAGCTCATTT TTTAACCAAT AGGCCGAAAT CGGCAAAATC
2281 CCTTATAAAT CAAAAGAATA GCCCGAGATA GGGTTGAGTG TTGTTCCAGT TTGGAACAAG
2341 AGTCCACTAT TAAAGAACGT GGACTCCAAC GTCAAAGGGC GAAAAACCGT CTATCAGGGC
2401 GATGGCCCAC TACGTGAACC ATCACCCAAA TCAAGTTTTT TGGGGTCGAG GTGCCGTAAA
2461 GCACTAAATC GGAACCCTAA AGGGAGCCCC CGATTTAGAG CTTGACGGGG AAAGCCGGCG
2521 AACGTGGCGA GAAAGGAAGG GAAGAAAGCG AAAGGAGCGG GCGCTAGGGC GCTGGCAAGT
2581 GTAGCGGTCA CGCTGCGCGT AACCACCACA CCCGCCGCGC TTAATGCGCC GCTACAGGGC
2641 GCGTACTATG GTTGCTTTGA CGTATGCGGT GTGAAATACC GCACAGATGC GTAAGGAGAA
2701 AATACCGCAT CAGGCGCCAT TCGCCATTCA GGCTGCGCAA CTGTTGGGAA GGGCGATCGG
2761 TGCGGGCCTC TTCGCTATTA CGCCAGCTGG CGAAAGGGGG ATGTGCTGCA AGGCGATTAA
2821 GTTGGGTAAC GCCAGGGTTT TCCCAGTCAC GACGTTGTAA AACGACGGCC AGTGAATTCG
2881 AATGGCCATG GAGGATTTAT TCTCTCCGCA GTTTTGAGCT TAATCGTCTC ATTAACTACT
2941 GCACTTTTCT CCGATTGGCT TCGTCGGAGA AAATAATCTA ACGGCATGAA GCGCAAATTA
3001 AAAGCCACTC AACTATGGGC CTAGTTGGGT GGCTTTTTTG CAACCATTAC GGTTTTACTT
3061 CTCTATAAGA ATTATAGCAT CAATCTTCAT CATTAGCCAA CTCAATAGAA GCTCGATATA
3121 GATCATGTTG AGCTTTATCC AAGCTATCTA AAAGTTCGCC ATAAATATCA ATATTTTTCT
3181 TTTCAGCAAC CAATTTTTTC AAATCTTGAA GATTATCAGC AATCTGTTCT GCCATAACTC
3241 CATAGATTTC AGCTTGTTTC TTCATATTGT CAATTGTCTT AATATCTTGT AACTCCATTC
3301 CTATCACTCC CATCTTACAT AGTTACTTAC CTTAGAATTC CAACGATATA TCACAGTATC
3361 TACAACATCA TCTTTTTCTT TTTCGTCATC CACATTAATT AGATCATCTT CTTTTTCGTC
3421 CAGCAATAAC TCATGATGAA TCTCTTTTAG TAATCCCGCA AAGCTTAATT GTCGAGTTCC
3481 AGCCAGAGCA TGCTCGAGCT CGTCAACAAC AACTAAATCG CCTTTCTCAT CATCCGTAAT
3541 ATAATCATAA TCTTTAACTT GATATTTACT TACTTCTTTT GCCGAAGCAA TCAAACTATT
3601 ATCTTTTTTT GACGCCCGAA TTTTCTTGAT ATTAACAATC GGCTTGTAGT CTAATTTCCT
3661 TGCTCGCTTC CATAATTGTG ACCAATTCTC TTGGGTAAGA TAATGACCCT TAGAAAAATA
3721 ACTAGATTTC ACCATTAACA AAATATGTAC ATGTTGATGG AAAGTTAGAC TATCTCGATT
3781 GACCGTTATT TCTGTAGATC GCACATAACC AAGAAGATCT TTTTTTACAT CTTTATATTG
3841 AAAAAGCCGA TAAATTGAAC GATTCATCGA AGTAAGATTT TTTCTAAGCT CGCCCAACTT
3901 TGAGCTTTCC TCAGTTAATG TCAAGAACAG AAATATAGCA CTAGGATCCT TTTTATATGC
3961 TGTATCTAAA ATTTGACGCA ACTCATAAGA ATTTTTCATC GATCGGCGCC AGCTACATAA
4021 TGGACAAAGT CTCGAATGAC AAAACCACGT TTGATATAAC TTTAAACCAT TATCAGTTTT
4081 CGCAAATTGA AGAACTTCTC CGCACTTTGA AACGTTATGA GCTTTTTTAA AATTTAAAAC
4141 CTCCAGATAT TGGGAATAAG TTAAGTTAGC AAGTTTTTTC TGCTTCCACG GTCTAACTTT
4201 CCCATTACTA GAGGTATCTT TTAGAATTTC TTTTTTATAC ATTTCTTATA CCTCTAACTG
4261 CAAAAACTAC AAGCAGTCAG AGTTAGTTAT CATTGATATA TTCCTAATGA CATATTATAA
4321 TAGGGTTCGA AGTTAATAAC GATAACTAAC TCATGCATCG ACGGCCAAAT CGAACAACTG
4381 CATGAGTTTT TTATTTAATT CTTACAATGC TTATTATACA CCAAAAAGTG TTATATTTTA
4441 AAATCCTAAT AAACCCAGTC ATATCAAGGG TTTAACCCAA TTTCAAAAAA TGGCGTTGTT
4501 TCTATATGTA TCAAGATAAG AAGAAACTCG CTTACAGCGA GTTTCTAAAT AGGGGTTTCA
4561 CCCCTAAAAC CCTGACCAGC TGAGCCAGCT GGACCGCATT TCCCCTCGCC GGGCGGGCGT
4621 AAAGCGGAAA AAATGACTTT TTTCTCGCTT TCGCTCAACT AGGGTCTTCC CGTCTCACCA
4681 TGTCAAGCGC TGCCCTTCCG CTACGCTCCA GGCGTTTGTC GCTTGACACT CACCTACTAA
4741 CAGCGGGCTT CGCCACCAGT CTACTATCTG CAACACGGCA ACAAGATTAA TCAGAATATG
4801 CATTGAAGCA GTGCGCTCCG CTCCTTGTCT TGCGACAAAG CGAGCGCACT GCTTCAATGC
4861 TTTTTTATTT TCTGAATTCC ATAACTCGAT AATTAGAAAA AGTTAGTCCG TTAGAAACGT
4921 CTGCCACGCC GAGGAAGTCA TTCCAGTTTG ACCACTCAAA TCATCAGCGA CATCATTGAG
4981 ATAATCAATC AGCAATTTTC TAGTTTCTAT CTGCTTTGGA ATTTTTACCA CAGCCAAAAT
5041 TGAGTCCCAC TTAGCAATCA CAAAAGATTT TCTGACGGCT TTATTAGCCT TTTCCGCACT
5101 AATATTTTTC GCTTTATCTC GTTTATTTTG ATCGGTCGAT GGTATCCGAT CGACTGTTGT
5161 TACAGCATTT GACGCAGGTT GCTAGATAAA CGACTAGACC GAATAATTTT TATTCCAACT
5221 ATTTTTGCAA CCAAAAAAAT CACAAAATAA TCAACCAATA AAACGATAAC TAACTTCTTG

5281 CTACATCCGA TTAATTTTTC TCCAGCCTGC CAGAAATCGG CCCAAATATT GAAATATTGA
5341 GGAAATTTTG GGCTGAAAAA AACATTAATT TTTTCGGCAA ACAAGCCAAC ACAGCCAAGC
5401 AGAAAAGCTA AGAAAATAAG TTCCAGCCAA TTAAAAAATC GCCACGAAAG AGAAAAAAAT
5461 CGGTCAAAAT AAATCGAACT TGATTTCATT TTCTAGCCCC CGAAGTCTTC GATAAACGGC
5521 GCCATAAAAG GAACTACACG GTTTTCCGGT TGAGTACTTG CAAGCCCTAA CCCCTTAGAA
5581 AAGCCGTCAT GATTAACAAC TGTTGTCGAC TCAGCTAGGG CCCTATATAT GGATCCAATT
5641 GCAATGATCA TCATGACAGA TCTGCGCGCG ATCGATATCA GCGCTTTAAA TTTGCGCATG
5701 CTAGCTATAG TTCTAGAGGT ACCGGTTGTT AACGTTAGCC GGCTACGTAT ACTCCGGAAT
5761 ATTAATAGGC CTAGGATGCA TATGCTTAGA AGCAAACTTA AGAGTGTGTT GATAGTGCAG
5821 TATCTTAAAA TTTTGTATAA TAGGAATTGA AGTTAAATTA GATGCTAAAA ATTTGTAATT
5881 AAGAAGGAGT GATTACATGA ACAAAAATAT AAAATATTCT CAAAACTTTT TAACGAGTGA
5941 AAAAGTACTC AACCAAATAA TAAAACAATT GAATTTAAAA GAAACCGATA CCGTTTACGA
6001 AATTGGAACA GGTAAAGGGC ATTTAACGAC GAAACTGGCT AAAATAAGTA AACAGGTAAC
6061 GTCTATTGAA TTAGACAGTC ATCTATTCAA CTTATCGTCA GAAAAATTAA AACTGAATAC
6121 TCGTGTCACT TTAATTCACC AAGATATTCT ACAGTTTCAA TTCCCTAACA AACAGAGGTA
6181 TAAAATTGTT GGGAGTATTC CTTACCATTT AAGCACACAA ATTATTAAAA AAGTGGTTTT
6241 TGAAAGCCAT GCGTCTGACA TCTATCTGAT TGTTGAAGAA GGATTCTACA AGCGTACCTT
6301 GGATATTCAC CGAACACTAG GGTTGCTCTT GCACACTCAA GTCTCGATTC AGCAATTGCT
6361 TAAGCTGCCA GCGGAATGCT TTCATCCTAA ACCAAAAGTA AACAGTGTCT TAATAAAACT
6421 TACCCGCCAT ACCACAGATG TTCCAGATAA ATATTGGAAG CTATATACGT ACTTTGTTTC
6481 AAAATGGGTC AATCGAGAAT ATCGTCAACT GTTTACTAAA AATCAGTTTC ATCAAGCAAT
6541 GAAACACGCC AAAGTAAACA ATTTAAGTAC CGTTACTTAT GAGCAAGTAT TGTCTATTTT
6601 TAATAGTTAT CTATTATTTA ACGGGAGGAA ATAATTCTAT GAGTCGCTTT TGTAAATTTG
6661 GAAAGTTACA CGTTACTAAA GGGAATGTAG ATAAATTATT AGGTATACTA CTGACAGCTT
6721 CCAAGCATAT GGCGGCCGCC TGCAGCTGGC GCCATCGATA CGCGTACGTC GCGACCGCGG
6781 ACATGTACAG AGCTCGAGAA GTACTAGTGG CCACGTGGGC CGTGCACCTT AAGCTTGGCG
6841 TAATCATGGT CATAGCTGTT TCCTGTGTGA AATTGTTATC CGCTCACAAT TCCACACAAC
6901 ATACGAGCCG GAAGCATAAA GTGTAAAGCC TGGGGTGCCT AATGAGTGAG CTAACTCACA
6961 TTAATTGCGT TGCGCTCACT GCCCGCTTTC CAGTCGGGAA ACCTGTCGTG CCAGCTGCAT
7021 TAATGAATCG GCCAACGCGC GGGGAGAGGC GGTTTGCGTA TTGGGCGC

pHENOMM

1 TCTTCCGCTT CCTCGCTCAC TGACTCGCTG CGCTCGGTCG TTCGGCTGCG GCGAGCGGTA
61 TCAGCTCACT CAAAGGCGGT AATACGGTTA TCCACAGAAT CAGGGGATAA CGCAGGAAAG
121 AACATGTGAG CAAAAGGCCA GCAAAAGGCC AGGAACCGTA AAAAGGCCGC GTTGCTGGCG
181 TTTTTCCATA GGCTCCGCCC CCCTGACGAG CATCACAAAA ATCGACGCTC AAGTCAGAGG
241 TGGCGAAACC CGACAGGACT ATAAAGATAC CAGGCGTTTC CCCCTGGAAG CTCCCTCGTG
301 CGCTCTCCTG TTCCGACCCT GCCGCTTACC GGATACCTGT CCGCCTTTCT CCCTTCGGGA
361 AGCGTGGCGC TTTCTCATAG CTCACGCTGT AGGTATCTCA GTTCGGTGTA GGTCGTTCGC
421 TCCAAGCTGG GCTGTGTGCA CGAACCCCCC GTTCAGCCCG ACCGCTGCGC CTTATCCGGT
481 AACTATCGTC TTGAGTCCAA CCCGGTAAGA CACGACTTAT CGCCACTGGC AGCAGCCACT
541 GGTAACAGGA TTAGCAGAGC GAGGTATGTA GGCGGTGCTA CAGAGTTCTT GAAGTGGTGG
601 CCTAACTACG GCTACACTAG AAGAACAGTA TTTGGTATCT GCGCTCTGCT GAAGCCAGTT
661 ACCTTCGGAA AAAGAGTTGG TAGCTCTTGA TCCGGCAAAC AAACCACCGC TGGTAGCGGT
721 GGTTTTTTTG TTTGCAAGCA GCAGATTACG CGCAGAAAAA AAGGATCTCA AGAAGATCCT
781 TTGATCTTTT CTACGGGGTC TGACGCTCAG TGGAACGAAA ACTCACGTTA AGGGATTTTG
841 GTCATGAGAT TATCAAAAAG GATCTTCACC TAGATCCTTT TAAATTAAAA ATGAAGTTTT
901 AAATCAATCT AAAGTATATA TGAGTAAACT TGGTCTGACA GTTACCAATG CTTAATCAGT
961 GAGGCACCTA TCTCAGCGAT CTGTCTATTT CGTTCATCCA TAGTTGCCTG ACTCCCCGTC
1021 GTGTAGATAA CTACGATACG GGAGGGCTTA CCATCTGGCC CCAGTGCTGC AATGATACCG
1081 CGAGACCCAC GCTCACCGGC TCCAGATTTA TCAGCAATAA ACCAGCCAGC CGGAAGGGCC

1141 GAGCGCAGAA GTGGTCCTGC AACTTTATCC GCCTCCATCC AGTCTATTAA TTGTTGCCGG
1201 GAAGCTAGAG TAAGTAGTTC GCCAGTTAAT AGTTTGCGCA ACGTTGTTGC CATTGCTACA
1261 GGCATCGTGG TGTCACGCTC GTCGTTTGGT ATGGCTTCAT TCAGCTCCGG TTCCCAACGA
1321 TCAAGGCGAG TTACATGATC CCCCATGTTG TGCAAAAAAG CGGTTAGCTC CTTCGGTCCT
1381 CCGATCGTTG TCAGAAGTAA GTTGGCCGCA GTGTTATCAC TCATGGTTAT GGCAGCACTG
1441 CATAATTCTC TTACTGTCAT GCCATCCGTA AGATGCTTTT CTGTGACTGG TGAGTACTCA
1501 ACCAAGTCAT TCTGAGAATA GTGTATGCGG CGACCGAGTT GCTCTTGCCC GGCGTCAATA
1561 CGGGATAATA CCGCGCCACA TAGCAGAACT TTAAAAGTGC TCATCATTGG AAAACGTTCT
1621 TCGGGGCGAA AACTCTCAAG GATCTTACCG CTGTTGAGAT CCAGTTCGAT GTAACCCACT
1681 CGTGCACCCA ACTGATCTTC AGCATCTTTT ACTTTCACCA GCGTTTCTGG GTGAGCAAAA
1741 ACAGGAAGGC AAAATGCCGC AAAAAAGGGA ATAAGGGCGA CACGGAAATG TTGAATACTC
1801 ATACTCTTCC TTTTTCAATA TTATTGAAGC ATTTATCAGG GTTATTGTCT CATGAGCGGA
1861 TACATATTTG AATGTATTTA GAAAAATAAA CAAATAGGGG TTCCGCGCAC ATTTCCCCGA
1921 AAAGTGCCAC CTGACGTCTA AGAAACCATT ATTATCATGA CATTAACCTA TAAAAATAGG
1981 CGTATCACGA GGCCCTTTCG TCTCGCGCGT TTCGGTGATG ACGGTGAAAA CCTCTGACAC
2041 ATGCAGCTCC CGGAGACGGT CACAGCTTGT CTGTAAGCGG ATGCCGGGAG CAGACAAGCC
2101 CGTCAGGGCG CGTCAGCGGG TGTTGGCGGG TGTCGGGGCT GGCTTAACTA TGCGGCATCA
2161 GAGCAGATTG TACTGAGAGT GCACCATAAA ATTGTAAACG TTAATATTTT GTTAAAATTC
2221 GCGTTAAATT TTTGTTAAAT CAGCTCATTT TTTAACCAAT AGGCCGAAAT CGGCAAAATC
2281 CCTTATAAAT CAAAAGAATA GCCCGAGATA GGGTTGAGTG TTGTTCCAGT TTGGAACAAG
2341 AGTCCACTAT TAAAGAACGT GGACTCCAAC GTCAAAGGGC GAAAAACCGT CTATCAGGGC
2401 GATGGCCCAC TACGTGAACC ATCACCCAAA TCAAGTTTTT TGGGGTCGAG GTGCCGTAAA
2461 GCACTAAATC GGAACCCTAA AGGGAGCCCC CGATTTAGAG CTTGACGGGG AAAGCCGGCG
2521 AACGTGGCGA GAAAGGAAGG GAAGAAAGCG AAAGGAGCGG GCGCTAGGGC GCTGGCAAGT
2581 GTAGCGGTCA CGCTGCGCGT AACCACCACA CCCGCCGCGC TTAATGCGCC GCTACAGGGC
2641 GCGTACTATG GTTGCTTTGA CGTATGCGGT GTGAAATACC GCACAGATGC GTAAGGAGAA
2701 AATACCGCAT CAGGCGCCAT TCGCCATTCA GGCTGCGCAA CTGTTGGGAA GGGCGATCGG
2761 TGCGGGCCTC TTCGCTATTA CGCCAGCTGG CGAAAGGGGG ATGTGCTGCA AGGCGATTAA
2821 GTTGGGTAAC GCCAGGGTTT TCCCAGTCAC GACGTTGTAA AACGACGGCC AGTGAATTCG
2881 AATGGCCATG GAGGATTTAT TCTCTCCGCA GTTTTGAGCT TAATCGTCTC ATTAACTACT
2941 GCACTTTTCT CCGATTGGCT TCGTCGGAGA AAATAATCTA ACGGCATGAA GCGCAAATTA
3001 AAAGCCACTC AACTATGGGC CTAGTTGGGT GGCTTTTTTG CAACCATTAC GGTTTTACTT
3061 CTCTATAAGA ATTATAGCAT CAATCTTCAT CATTAGCCAA CTCAATAGAA GCTCGATATA
3121 GATCATGTTG AGCTTTATCC AAGCTATCTA AAAGTTCGCC ATAAATATCA ATATTTTTCT
3181 TTTCAGCAAC CAATTTTTTC AAATCTTGAA GATTATCAGC AATCTGTTCT GCCATAACTC
3241 CATAGATTTC AGCTTGTTTC TTCATATTGT CAATTGTCTT AATATCTTGT AACTCCATTC
3301 CTATCACTCC CATCTTACAT AGTTACTTAC CTTAGAATTC CAACGATATA TCACAGTATC
3361 TACAACATCA TCTTTTTCTT TTTCGTCATC CACATTAATT AGATCATCTT CTTTTTCGTC
3421 CAGCAATAAC TCATGATGAA TCTCTTTTAG TAATCCCGCA AAGCTTAATT GTCGAGTTCC
3481 AGCCAGAGCA TGCTCGAGCT CGTCAACAAC AACTAAATCG CCTTTCTCAT CATCCGTAAT
3541 ATAATCATAA TCTTTAACTT GATATTTACT TACTTCTTTT GCCGAAGCAA TCAAACTATT
3601 ATCTTTTTTT GACGCCCGAA TTTTCTTGAT ATTAACAATC GGCTTGTAGT CTAATTTCCT
3661 TGCTCGCTTC CATAATTGTG ACCAATTCTC TTGGGTAAGA TAATGACCCT TAGAAAAATA
3721 ACTAGATTTC ACCATTAACA AAATATGTAC ATGTTGATGG AAAGTTAGAC TATCTCGATT
3781 GACCGTTATT TCTGTAGATC GCACATAACC AAGAAGATCT TTTTTTACAT CTTTATATTG
3841 AAAAAGCCGA TAAATTGAAC GATTCATCGA AGTAAGATTT TTTCTAAGCT CGCCCAACTT
3901 TGAGCTTTCC TCAGTTAATG TCAAGAACAG AAATATAGCA CTAGGATCCT TTTTATATGC
3961 TGTATCTAAA ATTTGACGCA ACTCATAAGA ATTTTTCATC GATCGGCGCC AGCTACATAA
4021 TGGACAAAGT CTCGAATGAC AAAACCACGT TTGATATAAC TTTAAACCAT TATCAGTTTT
4081 CGCAAATTGA AGAACTTCTC CGCACTTTGA AACGTTATGA GCTTTTTTAA AATTTAAAAC
4141 CTCCAGATAT TGGGAATAAG TTAAGTTAGC AAGTTTTTTC TGCTTCCACG GTCTAACTTT
4201 CCCATTACTA GAGGTATCTT TTAGAATTTC TTTTTTATAC ATTTCTTATA CCTCTAACTG
4261 CAAAAACTAC AAGCAGTCAG AGTTAGTTAT CATTGATATA TTCCTAATGA CATATTATAA
4321 TAGGGTTCGA AGTTAATAAC GATAACTAAC TCATGCATCG ACGGCCAAAT CGAACAACTG
4381 CATGAGTTTT TTATTTAATT CTTACAATGC TTATTATACA CCAAAAAGTG TTATATTTTA
4441 AAATCCTAAT AAACCCAGTC ATATCAAGGG TTTAACCCAA TTTCAAAAAA TGGCGTTGTT

4501 TCTATATGTA TCAAGATAAG AAGAAACTCG CTTACAGCGA GTTTCTAAAT AGGGGTTTCA
4561 CCCCTAAAAC CCTGACCAGC TGAGCCAGCT GGACCGCATT TCCCCTCGCC GGGCGGGCGT
4621 AAAGCGGAAA AAATGACTTT TTTCTCGCTT TCGCTCAACT AGGGTCTTCC CGTCTCACCA
4681 TGTCAAGCGC TGCCCTTCCG CTACGCTCCA GGCGTTTGTC GCTTGACACT CACCTACTAA
4741 CAGCGGGCTT CGCCACCAGT CTACTATCTG CAACACGGCA ACAAGATTAA TCAGAATATG
4801 CATTGAAGCA GTGCGCTCCG CTCCTTGTCT TGCGACAAAG CGAGCGCACT GCTTCAATGC
4861 TTTTTTATTT TCTGAATTCC ATAACTCGAT AATTAGAAAA AGTTAGTCCG TTAGAAACGT
4921 CTGCCACGCC GAGGAAGTCA TTCCAGTTTG ACCACTCAAA TCATCAGCGA CATCATTGAG
4981 ATAATCAATC AGCAATTTTC TAGTTTCTAT CTGCTTTGGA ATTTTTACCA CAGCCAAAAT
5041 TGAGTCCCAC TTAGCAATCA CAAAAGATTT TCTGACGGCT TTATTAGCCT TTTCCGCACT
5101 AATATTTTTC GCTTTATCTC GTTTATTTTG ATCGGTCGAT GGTATCCGAT CGACTGTTGT
5161 TACAGCATTT GACGCAGGTT GCTAGATAAA CGACTAGACC GAATAATTTT TATTCCAACT
5221 ATTTTTGCAA CCAAAAAAAT CACAAAATAA TCAACCAATA AAACGATAAC TAACTTCTTG
5281 CTACATCCGA TTAATTTTTC TCCAGCCTGC CAGAAATCGG CCCAAATATT GAAATATTGA
5341 GGAAATTTTG GGCTGAAAAA AACATTAATT TTTTCGGCAA ACAAGCCAAC ACAGCCAAGC
5401 AGAAAAGCTA AGAAAATAAG TTCCAGCCAA TTAAAAAATC GCCACGAAAG AGAAAAAAAT
5461 CGGTCAAAAT AAATCGAACT TGATTTCATT TTCTAGCCCC CGAAGTCTTC GATAAACGGC
5521 GCCATAAAAG GAACTACACG GTTTTCCGGT TGAGTACTTG CAAGCCCTAA CCCCTTAGAA
5581 AAGCCGTCAT GATTAACAAC TGTTGTCGAC TTTTTTTTGA TAAGTTAAGG GATGCAGTTT
5641 ATGCACCCCT TAACTTATCA TTATTAGTGT AAACAAGGAA GGATATCTTG TACGGCTTGA
5701 ACAATTACAC CACCAGCAGC GATATCAGCA GAAATACGAG CAATATGTTC GTCAAGTCCT
5761 TGTTCGTTAT CGTTCCAAAT ATATGGCCGA TCTGATGTAG GTTTTTGGCC GACAACATGA
5821 CGTACTGCTG AATAAAGACG TTCTGTAGCT GGACTTAAAC AAGCACGAGC ATCATAATGG
5881 CCCGTCTTTT TGTAAGTCCG TAAATCAACA GCTTGTACTC CAAACATTAA TGCAATAGCT
5941 ACGTAGTTTT GAAAAATATC AACAGACCGT CGTGCTAACG TAGCAGAAGT GTATCCTTGA
6001 GAGTTAATAT TTTGGTTAAA TTGTTCGGCG TGTGTAGGAA AGCGATCAGC AATTGAATTT
6061 CCGTAAAAAG TCAACAATGG CATAATTGAG TTGCCACAAA TTTGCAAACC CTTAAGACCC
6121 ATGTTTACCT TTCGTTCCCG GTTTCCCAAT AATGATGGTG GAAGTCCGTT ACTGAATTCT
6181 GGACTGGCCA AAAGTGCAAT TTGGACATCC AAATGCTTTG CAAGAAGACC AATGTAGTAA
6241 CGTAAGTGGT CCATGCCCAT GCCAACATAT TGACCCAAAA AGTTACCGCC ATGGTATGAG
6301 GCTTGGTTAT CAACGTCAAT TAATGGATTG TCAGTGACAC TATTAATTTC GATTTCAATT
6361 TGCTTAGCAA TTTGTGAAAT TCCATCAACA ATAGGACCCA AATATTGTGG TAAGCAACGT
6421 AAACTATAGC GGTCTTGAAT AAGTTCATGA TCACGATAAT CATGCTTACC ATCTAATTCG
6481 TCGCGAACAA GTTGACTATT AGCTAAAAGA GAAATCATTT GATCAGCAGC CCAAAGTTGA
6541 CCTGGGTGAG GTTTAGAATT GTGAATGAAT GGGTGGAAAC TTTGATTTGT GCCATTAAGT
6601 GCTTGAATAT CTAAGGCATG AACACCCATG GCAATAGCAG TAAGAATTTG AGTATCATAA
6661 ACGCAATTAG CAGCAATTCC CGTCATAACT GATGTACCAT TCATCATAGC AAGACCTTCT
6721 TTTGGAAGTA ATGTAAGTGG TGATAAATTT AATTGTCGAA GAGCTGTTGG GGCATCCATT
6781 TCTTTACCAT TAAAATCAAC TTTGAATGAT GGATCCAAAC CAATTAAAGA ACCTGTAATA
6841 TAAGATAAAG GAACAAGATC TCCACTTGCA CCAATTGATC CGAATTCGTA AACATAAGGT
6901 GTAACACCGG CGTTAAGGAA AATTTCCATA CGCTTAATTA ATTCAAGACG AATACCAGAA
6961 GCGCCACGCA TGTGTGAATT TGCACGTAAT AACATAGCAG CACGAACATC TGCTAATGGA
7021 AGTTTATTAC CAGCTCCTGT TTTAAGAAAC CAAACAAGAT TAGTTTGAAG TTCAGAAGCT
7081 TGTTCACGAC TAATAGCGAC GTTTGCCATT CCACCGAATC CTGAAGTAAC ACCATAAATT
7141 GGTTCTCCTG ATTCAACAGC GTTATTGATA TAGTCGCAAC TAGCTTGAAT GCCTTGTAAA
7201 ATGTCAGTGT TGTTAGTTAA ACTAACTAAA GTACCATTGC GTGCAACCCG TGCAACATCG
7261 TTAATAGTAA GCTTTTGATT ACCAATGATA ACATTAGCAC TTGAGTTTCC AGTAAATGAG
7321 AATTGTTGAG AACTAGTCTT AGATTGTGCT TGAGATAAAG TCTTCATTTA TACCTACTCC
7381 TTCTTAATTA CAAATTTTTA GCATCTAATT TAACTTCAAT TCCTATTATA CAAAATTTTA
7441 AGATACTGCA CTATCAACAC ACTCTTGAAC ATATGCATCC TAGGTCGTCT ATTAATACTT
7501 CCAGGAGTAT ACGTAGCCAG GCTAACGTTA ATCAACCAGG TATCCTCTAG AACTATAGCT
7561 AGCATGCCTA TATATGGATC CAATTGCAAT GATCATCATG ACAGATCTGC GCGCGATCGA
7621 TATCAGCGCT TTAAATTTGC GCATGCTAGC TATAGTTCTA GAGGTACCGG TTGTTAACGT
7681 TAGCCGGCTA CGTATACTCC GGAATATTAA TAGGCCTAGG ATGCATATGC TTAGAAGCAA
7741 ACTTAAGAGT GTGTTGATAG TGCAGTATCT TAAAATTTTG TATAATAGGA ATTGAAGTTA
7801 AATTAGATGC TAAAAATTTG TAATTAAGAA GGAGTGATTA CATGAACAAA AATATAAAAT

7861 ATTCTCAAAA CTTTTTAACG AGTGAAAAAG TACTCAACCA AATAATAAAA CAATTGAATT
7921 TAAAAGAAAC CGATACCGTT TACGAAATTG GAACAGGTAA AGGGCATTTA ACGACGAAAC
7981 TGGCTAAAAT AAGTAAACAG GTAACGTCTA TTGAATTAGA CAGTCATCTA TTCAACTTAT
8041 CGTCAGAAAA ATTAAAACTG AATACTCGTG TCACTTTAAT TCACCAAGAT ATTCTACAGT
8101 TTCAATTCCC TAACAAACAG AGGTATAAAA TTGTTGGGAG TATTCCTTAC CATTTAAGCA
8161 CACAAATTAT TAAAAAAGTG GTTTTTGAAA GCCATGCGTC TGACATCTAT CTGATTGTTG
8221 AAGAAGGATT CTACAAGCGT ACCTTGGATA TTCACCGAAC ACTAGGGTTG CTCTTGCACA
8281 CTCAAGTCTC GATTCAGCAA TTGCTTAAGC TGCCAGCGGA ATGCTTTCAT CCTAAACCAA
8341 AAGTAAACAG TGTCTTAATA AAACTTACCC GCCATACCAC AGATGTTCCA GATAAATATT
8401 GGAAGCTATA TACGTACTTT GTTTCAAAAT GGGTCAATCG AGAATATCGT CAACTGTTTA
8461 CTAAAAATCA GTTTCATCAA GCAATGAAAC ACGCCAAAGT AAACAATTTA AGTACCGTTA
8521 CTTATGAGCA AGTATTGTCT ATTTTTAATA GTTATCTATT ATTTAACGGG AGGAAATAAT
8581 TCTATGAGTC GCTTTTGTAA ATTTGGAAAG TTACACGTTA CTAAAGGGAA TGTAGATAAA
8641 TTATTAGGTA TACTACTGAC AGCTTCCAAG CATATGGCGG CCGCCTGCAG CTGGCGCCAT
8701 CGATACGCGT ACGTCGCGAC CGCGGACATG TACAGAGCTC GAGAAGTACT AGTGGCCACG
8761 TGGGCCGTGC ACCTTAAGCT TGGCGTAATC ATGGTCATAG CTGTTTCCTG TGTGAAATTG
8821 TTATCCGCTC ACAATTCCAC ACAACATACG AGCCGGAAGC ATAAAGTGTA AAGCCTGGGG
8881 TGCCTAATGA GTGAGCTAAC TCACATTAAT TGCGTTGCGC TCACTGCCCG CTTTCCAGTC
8941 GGGAAACCTG TCGTGCCAGC TGCATTAATG AATCGGCCAA CGCGCGGGGA GAGGCGGTTT
9001 GCGTATTGGG CGC
