# Supplementary figures and images for: Genetically engineered probiotic for the treatment of phenylketonuria (PKU); assessment of a novel treatment in vitro and in the PAHenu2 mouse model of PKU
Source: PLoS One. 2017 May 17;12(5):e0176286. doi: 10.1371/journal.pone.0176286 (PMC5435137; doi:10.1371/journal.pone.0176286)

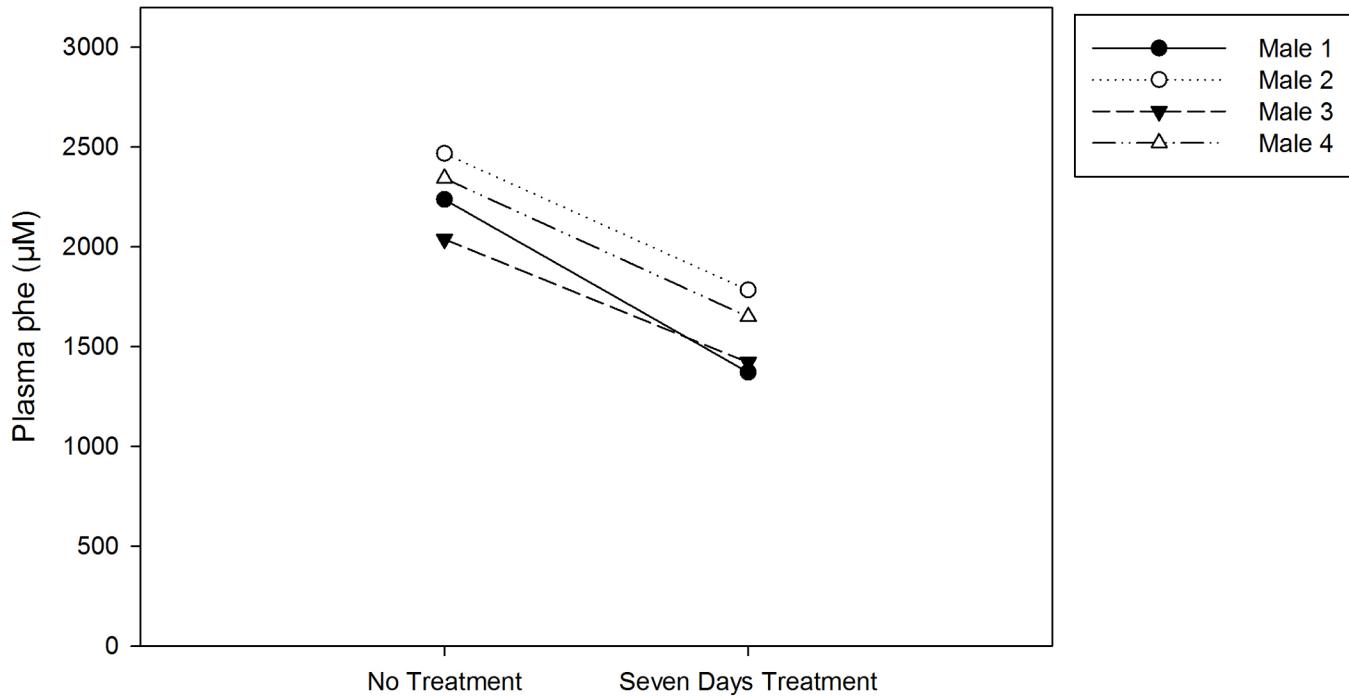

Supplement: S1 Fig — Four male animals were used for this experimental run with blood collected pre treatment and after seven days of treatment with pHENOMMenal probiotic. Mean plasma phe decrease was 715.1 ± 106.5μM, P < 0.0005. (PDF) [file pone.0176286.s002.pdf]

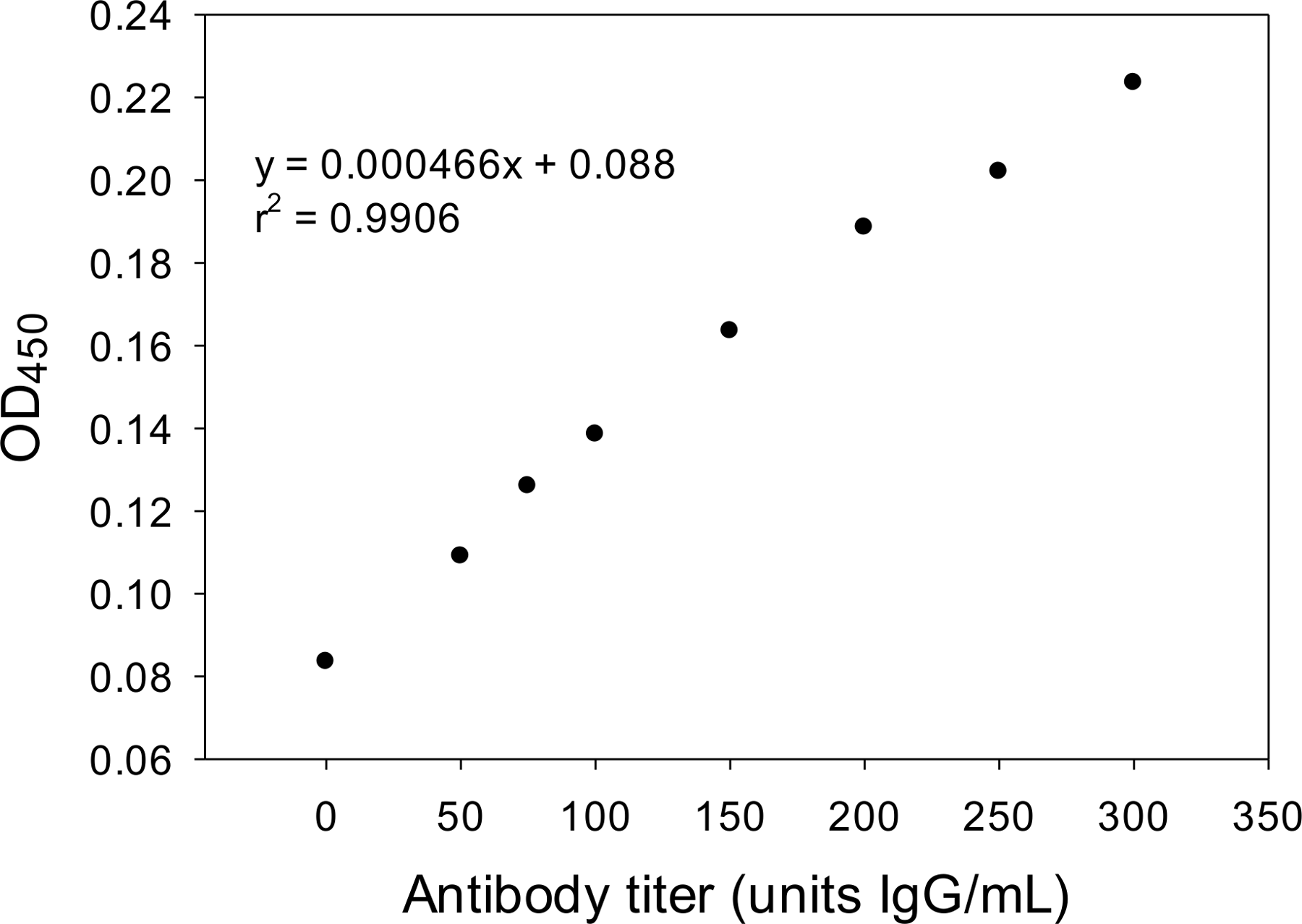

Supplement: S2 Fig — (TIF) [file pone.0176286.s003.tif]
